# UBR5 promotes tumor immune evasion through enhancing IFN-γ-induced *PDL1* transcription in triple negative breast cancer

**DOI:** 10.7150/thno.74989

**Published:** 2022-07-04

**Authors:** Bingbing Wu, Mei Song, Qun Dong, Gang Xiang, Jing Li, Xiaojing Ma, Fang Wei

**Affiliations:** 1 Sheng Yushou Center of Cell Biology and Immunology, Joint International Research Laboratory of Metabolic & Developmental Sciences, School of Life Science and Biotechnology, Shanghai Jiao Tong University, Shanghai, China.; 2 Department of Microbiology and Immunology, Weill Cornell Medicine, New York, New York.; 3 Department of Bioinformatics and Biostatistics, School of Life Sciences and Biotechnology, Shanghai Jiao Tong University, Shanghai, China

**Keywords:** UBR5, Interferon-γ, PD-L1, PKR/STAT1/IRF1, triple negative breast cancer

## Abstract

**Background:** The up-regulation of PD-L1 is recognized as an adaption of cancer cells to evade immune surveillance and attack. However, the intrinsic mechanisms of the induction of PD-L1 by interferon-γ (IFN-γ) in tumor microenvironment remain incompletely characterized. Ubiquitin ligase E3 component N-recognition protein 5 (UBR5) has a critical role in tumorigenesis of triple negative breast cancer (TNBC) by triggering specific immune responses to the tumor. Dual targeting of UBR5 and PD-L1 exhibited superior therapeutic benefits in a preclinical TNBC model in short term.

**Methods:** The regulation of UBR5 to PD-L1 upon IFN-γ stimulation was evaluated through in UBR5 deficiency, reconstitution or overexpression cell line models by quantitative PCR, immunohistochemistry and RNA-seq. The effects of PD-L1 regulation by UBR5 and double blockade of both genes were evaluated in mouse TNBC model. Luciferase reporter assay, chromatin immunoprecipitation-qPCR and bioinformatics analysis were performed to explore the transcription factors involved in the regulation of UBR5 to PD-L1.

**Results:** E3 ubiquitin ligase UBR5 plays a key role in IFN-γ-induced *PDL1* transcription in TNBC in an E3 ubiquitination activity-independent manner. RNA-seq-based transcriptomic analyses reveal that UBR5 globally affects the genes in the IFN-γ-induced signaling pathway. Through its poly adenylate binding (PABC) domain, UBR5 enhances the transactivation of* PDL1* by upregulating protein kinase RNA-activated (PKR), and PKR's downstream factors including signal transducers and activators of transcription 1 (STAT1) and interferon regulatory factor 1 (IRF1). Restoration of PD-L1 expression in UBR5-deficient tumor cells recoups their malignancy *in vivo*, whereas CRISPR/Cas9-mediated simultaneous abrogation of UBR5 and PD-L1 expression yields synergistic therapeutic benefits than either blockade alone, with a strong impact on the tumor microenvironment.

**Conclusions:** This study identifies a novel regulator of *PDL1* transcription, elucidates the underlying molecular mechanisms and provides a strong rationale for combination cancer immunotherapies targeting UBR5 and PD-L1.

## Introduction

Tumor cells can adapt immune regulatory signaling pathways to evade immune recognition and elimination. One of the mechanisms utilized by tumor cells is the upregulation of PD-L1, which has been identified as an indicator of poor prognosis in various tumor types, including breast cancer 1, 2. It has been reported that the mRNA and protein levels of PD-L1 are elevated in triple negative breast cancer (TNBC) cells 3. In breast cancers, higher expression level of PD-L1 is associated with larger tumor size, higher tumor grade, and increased positive lymph node number 4, 5. The PD-1/PD-L1 interaction can cause T cells to enter a state of anergy/exhaustion, which manifests impaired active proliferation, cytokine production and cytotoxicity 6, 7. Thus, targeting the PD-1/PD-L1 axis is a beneficial approach in the treatment of different cancers. Blockade of PD-1 or PD-L1 with monoclonal antibodies can reverse many of these phenomena and restore T cell function 8. Recently, atezolizumab and pembrolizumab showed durable antitumor activity as first-line therapies for patients with PD-L1-positive TNBC by blocking the interaction between PD-1 and its ligand PD-L1 9, 10. However, the patient response rate was still lower than expected. Thus, a better understanding of PD-L1 regulation may help predict patient responses and improve treatment.

The expression of PD-L1 can be exogenously induced by various cytokines including interferon-γ (IFN-γ), tumor necrosis factor-α (TNF-α), interleukins (ILs) and epidermal growth factor (EGF) through the Janus kinase (JAK)/signal transducer and activator of transcription 1 (STAT1)/interferon regulatory factor1 (IRF1), nuclear factor‑κB (NF-κB), phosphatidylinositol 3-kinase (PI3K)/AKT/mammalian target of rapamycin (mTOR) or JAK/STAT3 signaling pathways 11, 12. Among these factors, IFN-γ in the tumor microenvironment (TME) affects both tumor and immune cells in both immunoactivating and immunosuppressive ways 13 which explains why that early approaches targeting IFN-γ in the TME largely failed to provide any clinical benefit 14-16. IFN-γ-induced adaptive immune resistance highlights the importance of utilizing IFN-γ-mediated immunotherapies by simultaneously blocking the expression or activity of PD-L1 and other factors 13. However, the intrinsic mechanism controlling IFN-γ-induced *PDL1* transcription remains incompletely characterized.

Human ubiquitin protein ligase E3 component N-recognin 5 (UBR5) was originally identified in a screen for progestin-regulated genes in breast cancer cells 17. *UBR5*, a member of a rare “homologous to E6-AP C-terminus” (HECT)-domain E3 ubiquitin ligase family 17, is highly conserved in metazoans and is essential for early embryonic development in mice 18, 19. UBR5 is frequently amplified and overexpressed in many cancer types, especially in human breast cancer and ovarian cancer 20, 21. Our previous work revealed a critical role of UBR5 in the aggression of a murine TNBC model 21. Overexpression of UBR5 was shown to correlate with poor overall survival in breast cancer 22. Two key functional domains, the HECT and poly adenylate binding C terminal (PABC) domains of UBR5, are well characterized. The HECT domain associates mainly with E3 ligase activity, and the PABC domain is thought to be a protein-protein interaction motif 23, 24 and may regulate ubiquitin transfer catalyzed by the HECT domain 25. Frameshift mutations tend to occur in the PABC/HECT domain in tumors 22. UBR5 has been reported to directly interact with various proteins implicated in a wide variety of cellular processes, including cell cycle, transcriptional and translational machinery, and DNA damage response. Known targets of UBR5 ligase activity include β-catenin 26, pregnane X receptor 27, and E6-AP 28.

We previously reported that UBR5-deficiency can facilitate the processing and presentation of tumor antigens by antigen-presenting cells to host T cells, triggering specific immune responses to the tumor 21. Dual targeting of UBR5 and PD-L1 exhibited superior therapeutic benefits in a preclinical TNBC model in short term 29. Here, we report for the first time that UBR5 globally regulates IFN-γ-mediated pathways and stimulated genes, particularly PD-L1 expression and uncover the underlying molecular mechanism. We also showed here that simultaneous abrogation of *Ubr5* and *Pdl1* expression has synergistic therapeutic benefits in long term.

## Materials and Methods

### Cell lines

Murine TNBC cell lines 4T1, and its derivative 4T1/GFP, 4T1/*Ubr5^-/-^*, human TNBC cell lines BT549, MDA-MB-231, ER^+^ breast carcinoma cell lines MCF7 and human embryonic kidney cell lines HEK293T were stored in Ma lab at SJTU. 4T1 cells were cultured with RPMI 1640 (Invitrogen) containing 10% fetal bovine serum (BI) and 100 μg/mL penicillin/streptomycin (Invitrogen). BT549, MDA-MB-231, MCF7 and HEK293T cells were maintained in DMEM (Invitrogen) supplemented with 10% FBS (BI) and 100 μg/mL penicillin/streptomycin (Invitrogen). All cell lines were incubated under an atmosphere of 5% CO_2_ at 37℃.

### Mice and mouse tumor model

Wild-type **(**BALB/c**)** female mice (6-8 weeks old) were purchased from the Charles River Laboratories (Pinghu, China) and maintained in a pathogen-free facility, supplied with sterile food and water.

For 4T1 tumor model, 1×10^6^ cells were injected into the 4^th^ mammary fat pads of BALB/c mice. Tumor growth was measured every 3 days with a caliper and tumor volume was calculated as volume=1/2×length×width^2^. For lung metastasis experiment, 5×10^5^ 4T1 cells were suspended in 100 μL PBS and then intravenously injected into BALB/c mice through the tail vein. Twelve days later, mice were sacrificed and the lungs were collected and made single cell suspension to perform tumor cell metastasis assay *in vitro* with 6-thioguanine as described previously 21.

### Plasmids and vectors

For the constructs used in luciferase reporter assay, mouse *Pdl1* and human* PDL1* promoters were amplified by PCR from genomic DNA isolated from 4T1 or BT549 cells using the primers listed in **Table S1**, then cloned into the multicloning site (MCS) of the pGL3 Basic Vector (Promega Corporation). Specific deletions of the putative binding sites were carried out by the protocol described elsewhere 11, with primers listed in **Table S1**. For UBR5 mutant UBR5-ΔPABC, 78 amino acids of the PABC domain were deleted using overlapping PCR. The m*Eif2ak2* cDNA were amplified by PCR from 4T1 cells cDNA pool using primers listed in Table S1. The RNA interference (RNAi) from a lentiviral vector were generated with specific short hairpin RNA (shRNA) expression for each gene. All shRNA sequences were listed in **Table S2**.

### CRISPR/Cas9-mediated knockout

4T1 and its derivative cells were subjected to CRISPR/Cas9-mediated knockout of *Pdl1* by transient transfection of lentiCRISPR v2 based vector carrying the guide sequences specific for PD-L1. Three guide sequences used per gene were listed in **Table S2**. Positive single-cell clones were screened using 4 μg/mL puromycin. Disruption was confirmed finally by western blot and FACS analysis.

### Cell transfections and infections

For the reconstitution of human *UBR5* or mouse *Pdl1*, 4T1/*Ubr5^-/-^* cells were transfected with h*UBR5* and m*Pdl1* plasmids by Lipofectamine 2000 reagent (Invitrogen). Stable h*UBR5* or m*Pdl1* reconstited-4T1/*Ubr5^-/-^* cells were obtained by transfecting plasmids pCDH-h*UBR5* or pCDH-m*Pdl1* and selecting by puromycin. Western blot or FACS were used to confirm the efficiency of reconstitution. 4T1/GFP cells were transiently transfected with siRNA targeting JAK3, STAT1, STAT2, IRF1, and IRF7 individually by Lipofectamine 2000 reagent. siNC (non-target control) was used as the negative control. All siRNA were designed and purchased from GenePharm. Lentiviruses were produced by cotransfection of 293T cells with PSPA, pMD2G, and pGIPZ-dtTomato-shUBR5 or shEIF2AK2 with polyethyleneimine (PEI). Virus supernatants were collected at 24 h and 48 h post-transfection. MDA-MB-231 and BT549 cells were infected with shUBR5 containing lentivirus, then were selected with puromycin. 4T1 cells were infected with shEIF2AK2 containing lentivirus and treated with puromycin. A scrambled shRNA was used as the negative control (shNC).

### Quantitative RT-PCR (RT-qPCR)

Total RNA was extracted with Trizol (Invitrogen) and cDNA was synthesized using the cDNA Synthesis Kit (Vazyme). RT-qPCR was performed with Hieff qPCR SYBR Green Master Mix (YEASEN). The cDNA was quantified using SYBR mRNA expression assays by CFX96 Touch Real-Time PCR Detection System (Bio-Rad). The primers sequence of target genes were listed in **Table S3**.

### Western blot

Cells were lysed in RIPA buffer (Beyotime Biotechnology) on ice for 10 min. Cell lysates were centrifuged at 12,000 rpm for 15 min at 4℃, and supernatant was collected. Protein concentration was quantified by Beyotime protein assay (Beyotime Biotechnology, 5000006). Proteins were resolved on a 10% SDS PAGE gel and transferred to the NC membrane, blocked with 5% milk and probed for monoclonal antibodies against UBR5 (Santa Cruz, sc-515494), PD-L1 (Proteintech) #66248-1-lg, EIF2AK2 (Beyotime) #AF2125, STAT1 p84/91 (C-136) (Santa Cruz, sc-464), STAT1 (D1K9Y) Rabbit (Cell Signal Technology) mAb #14994, Phospho-STAT1 (Tyr701) Rabbit (Beyotime) #AF5935, IRF1 (E-4) (Santa Cruz, sc-514544) or anti-GAPDH (Proteintech).

### Flow cytometry analysis

For IFN-γ stimulation and PD-L1 staining in cell lines, TNBC cell lines were seeded into 6-well plates on Day 1, targeting 70-80% of confluence on the day of surface staining. On Day 2, cells were treated with 10 ng/mL IFN-γ (mouse IFN-γ: Sino Biological #50709-MNAH; human IFN-γ: PEPROTECH #300-02) for 24 h. On Day 3, cells were trypsinized and stained with allophycocyanin (APC) labelled anti-PD-L1 antibodies (Biolegend) on ice for 30 min, then washed with staining buffer for three times. For single cell staining, cells were dissociated from tumors or lymph nodes, then stained with antibodies accordingly. Antibodies against CD4 (RM4-5), CD3 (17A2), CD8 (53-6.7), Foxp3 (150D), CD25 (PC61), Granzyme B (GB11), and IFN-γ (XMG1.2) were purchased from Biolegend. Antibodies against CD45 (30-F11), CD11c (HL3), and MHCⅡ (2G9) were purchased from eBioscience. Flow cytometry data were analyzed by FlowJo software.

### Quantification of the micrometastases in lungs

To quantify micrometastases, mice were sacrificed twelve days after 5×10^5^ 4T1 cells injected into mice through i.v. Lungs were excised, minced, and digested with tissue dissociation buffer [0.25% collagenase IV (384 unit/mg, Worthington), 0.2% Dipase II (Roche), and 0.01% DNase I (Sigma) in PBS] with periodic votexing for 1 h in 37℃ water bath. Single cell suspension was washed and strained with 70 mm strainer, then plated in 60 μmol/L 6-thioguanine selection (serve as duplicates). After 1 to 2 weeks of selection, tumor colonies were stained with crystal violet for 10 min, rinsed with ultrapure water and dried overnight prior to counting.

### RNA-seq analysis

Total RNA was isolated for RNA-seq analysis. Second generation of RNA sequencing was performed by Genomic Core Facilities at Weill Cornell Medicine. High-quality reads were aligned to the mouse reference genome (vM25) using Histat2. We next used the featureCounts function of the subread software package to count the number of reads that mapped to a reference gene and performed differential expression with DEseq2. The enrichment analyses were based on differential expressed genes (padj <0.05, |foldchange|>2) using clusterProfiler R package.

### Bioinformatic analysis of TCGA database

The KEGG pathway analysis was used by R clusterProfiler. Based on the data of all cancer expression profiles (FPKM) of TCGA, Pearson Correlation was used to calculate pairwise expression correlations between UBR5 and ISGs.

### Transient luciferase reporter assays

Cells were seeded in 6-well plates for 18 h and then transfected using Lipofectamine 2000 reagent with 0.5 µg plasmid each (0.05 µg Renilla DNA was used for normalization). Cells were then treated with or without 10 ng/mL IFN-γ. 24 h later, relative luciferase units (RLUs) were measured using the Dual-Luciferase Report Assay System and GloMax 96 Microplate Luminometer (Promega) according to the manufacturer's instructions. RLUs from firefly luciferase signal were normalized by RLUs from Renilla signal.

### ChIP-qPCR

Formaldehyde-cross-linked chromatin was prepared from 1×10^7^ WT, *Ubr5^-/-^* and h*UBR5*-reconstituted *Ubr5^-/-^* 4T1 cells, and chromatin immunoprecipitation (ChIP) was performed using the EZ ChIP Kit (#17-371) from Millipore according to the manufacturer's instructions. Normal Mouse immunoglobulin G (IgG, sc-2025), anti-STAT1 p84/91 (C-136) (sc-464) and anti-IRF1 (E-4) (sc-514544) antibodies were purchased from Santa Cruz. Normal rabbit IgG (#2729) was purchased from Cell Signal Technology. Anti-H3K27ac (ab4729) antibody was purchased from Abcam. Anti-H3K4me1 antibody (#39498) was purchased from Active Motif. Real-time qPCR was performed in a CFX96 real-time PCR system (Bio-Rad) using Hieff qPCR SYBR Green Master Mix (YEASEN) and the primers for the GAPDH and PD-L1 promoters were listed in **Table S4**.

### Statistical analysis

All values were presented as mean ± SEM, and the Student t test was used to determine statistical differences between groups. Values of P < 0.05 were considered statistically significant. These analyses were carried out using the GraphPad Prism 6 for statistical software.

## Results

### UBR5 is required for IFN-γ-induced *PDL1* gene expression

**Figure 1:**
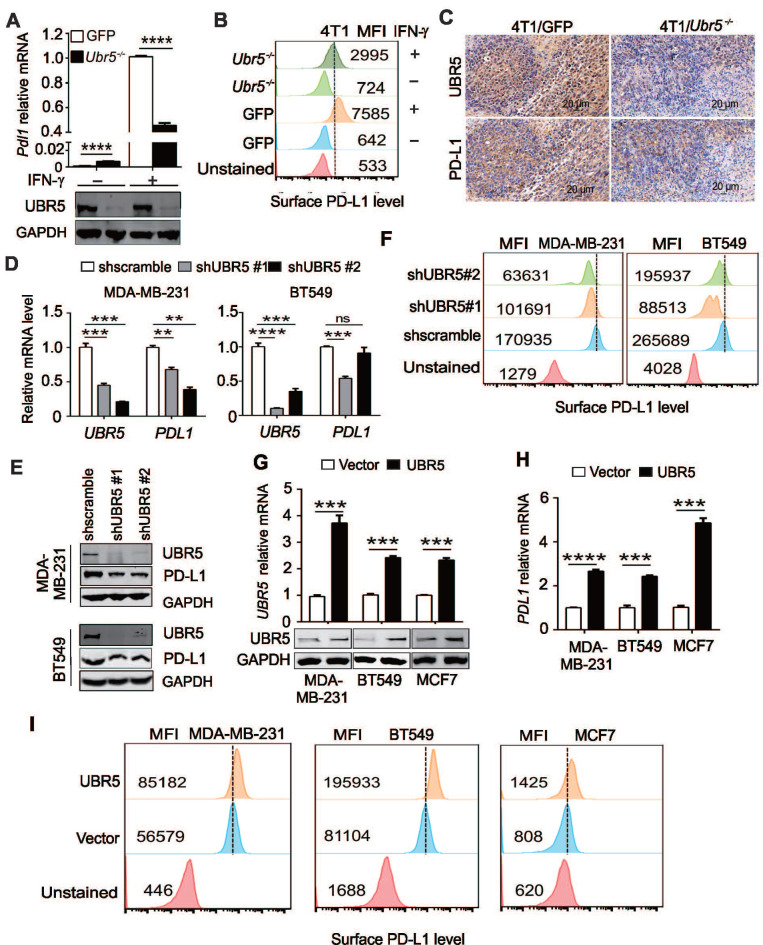
** UBR5 is required for IFN-γ-induced *PDL1* gene expression.** (A) Quantitative PCR analysis of the relative *Pdl1* mRNA levels and western blot analysis of the UBR5 protein levels in WT and *Ubr5^-/-^* 4T1 cells with or without 10 ng/mL IFN-γ treatment for 24 h. (B) The relative expression levels of PD-L1 on the surfaces of WT and *Ubr5^-/-^* 4T1 cells with or without 10 ng/mL IFN-γ stimulation was evaluated by flow cytometry. (C) WT and *Ubr5^-/-^* 4T1 cells (1×10^6^) were subcutaneously injected into the mammary pads to generate tumors in WT (BALB/c) mice. Immunohistochemical staining was performed with anti-UBR5 or anti-PD-L1 antibody in WT and *Ubr5^-/-^* 4T1 tumor sections. (D-F) Human TNBC BT549 and MDA-MB-231 cell lines with stable UBR5 knockdown were generated by infection with a lentivirus containing a control or UBR5-targeted shRNA sequence followed by puromycin selection. The mRNA (D) and protein levels of UBR5 (E) and PD-L1 (E and F) were measured after cells were treated with 10 ng/mL IFN-γ for 24 h.(G-I) BT549, MDA-MB-231 and MCF7 cells were transfected with an empty vector or UBR5 plasmids. 48 hours later, the cells were treated with 10 ng/mL IFN-γ for 24 h. The mRNA and protein levels of UBR5 (G) and PD-L1 (H and I) were measured by qPCR, western blot and FACS. All experiments were repeated at least three times, and the data are presented as the mean ± SEM. ns, no significance, **P < 0.01, ***P < 0.001, ****P < 0.0001.

Tumor cells can respond to elevated IFN-γ levels in the tumor microenvironment by upregulating the expression of PD-L1 to evade immune surveillance 11. Interestingly, we observed that the IFN-γ-mediated induction of PD-L1 in previous generated 29 4T1/*Ubr5^-/-^* cells (**Figure 1A**) was weaker (~50%) than that in control 4T1/GFP cells at both the mRNA and protein levels (**Figure 1A**-**B**). To determine whether the same phenomenon occurs *in vivo*, 4T1/GFP and 4T1/*Ubr5^-/-^* cells were subcutaneously injected into the 4^th^ mammary fat pads of BALB/c mice individually, and tumors were dissected at Day 28 after injection. Immunohistochemistry staining data (**Figure 1C**) showed that the PD-L1 levels were lower in 4T1/*Ubr5^-/-^* than in 4T1/GFP tumors.

To confirm that the effect of UBR5 on IFN-γ-induced PD-L1 expression was not due to cell line specificity, we evaluated the expression level of PD-L1 in short hairpin RNA (shRNA)-mediated *UBR5* knockdown MDA-MB-231 and BT549 cells. Upon IFN-γ stimulation, only 32-54% and 49-66% of *PDL1* mRNA and protein were expressed in *UBR5* knockdown MDA-MB-231 and BT549 cells compared with scramble control cells, which was consistent with the finding in 4T1 cells (**Figure 1D-E**). The PD-L1 surface protein levels in these cells were correlated with the mRNA levels (**Figure 1F**). Reciprocally, overexpression of UBR5 in BT549, MDA-MB-231 and MCF7 cell lines increased IFN-γ-induced PD-L1 expression at both the mRNA and surface protein levels (**Figure 1G, H-I**). Taken together, the findings indicate that there is a strong correlation between UBR5 and IFN-γ-stimulated PD-L1 expression.

Next, to explore whether the positive correlation between UBR5 and PD-L1 exists in other cancers beyond breast cancer, we evaluated the correlation between UBR5 and PD-L1 in TCGA database. TCGA-based analyses also highlight that the mRNA expression levels of *UBR5* and *PDL1* are highly correlated in many other cancer types, such as pancreatic adenocarcinoma (PAAD), thymoma (THYM), uveal melanoma (UVM), and prostate adenocarcinoma (PRAD) (**Figure S1**).

### Restoration of PD-L1 expression in UBR5-deficient tumor regains malignancy

**Figure 2:**
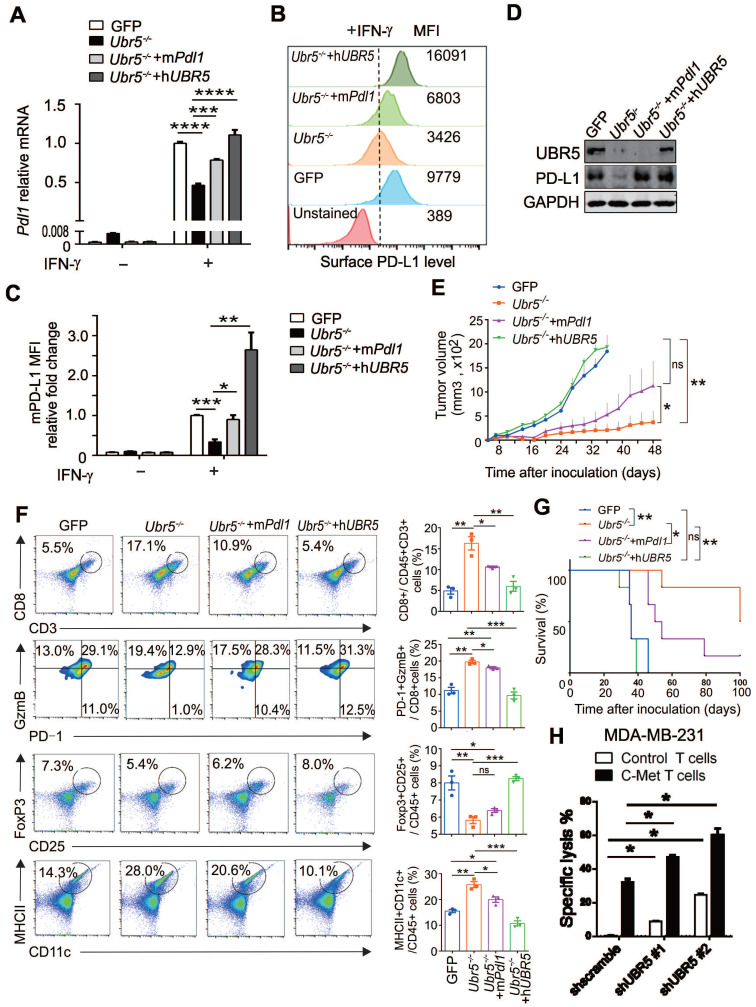
** Restoration of PD-L1 expression in UBR5-deficient tumors reinvigorates malignancy.** (A-D) *Ubr5^-/-^* 4T1 cells were transfected with a plasmid encoding m*Pdl1,* and then subjected to 4 μg/mL puromycin selection to generate stable m*Pdl1*-reconstituted 4T1/*Ubr5^-/-^* cells. The mRNA and protein levels of PD-L1 and UBR5 protein levels in WT, *Ubr5^-/-^*, h*UBR5-* or m*Pdl1*-reconstituted *Ubr5^-/-^* 4T1 cells were confirmed by RT-qPCR (A), FACS analysis (B) and western blot (D) after treatment with IFN-γ for 24 h, and the relative fold changes in PD-L1 based on the mean fluorescence intensity (MFI) are shown in (C). The data are presented as the mean ± SEM (error bar) from three replicates. *P< 0.05, **P < 0.01, ***P < 0.001, ****P < 0.0001. (E) A total of 1×10^6^ WT, *Ubr5^-/-^*, h*UBR5-* or m*Pdl1*-reconstituted *Ubr5^-/-^* 4T1 cells were subcutaneously injected into the mammary pads of WT (BALB/c) mice (n=6 mice per group). Tumor size was monitored every 3 days. (F) On Day 10 after tumor cells inoculation, flow cytometry analyzed the CD8^+^ T cells infiltration and GzmB^+^/PD-1^-^/CD8^+^ T cells in tumor tissue from mice bearing 4T1 WT, *Ubr5^-/-^*, h*UBR5-* or m*Pdl1*-reconstituted *Ubr5^-/-^* tumor. Tumor-draining lymph nodes were analyzed by staining for CD25^+^, Foxp3^+^ Tregs and CD11c^+^, MHC Ⅱ^+^ DCs by flow cytometry. The results are presented as the mean ± SEM. ns, no significance, *P< 0.05, **P < 0.01, ***P < 0.001. n=3 mice per group. Mouse survival (G) were recorded daily. (H) T cells cytotoxicity difference toward MDA-MB-231 cells with different UBR5 expression levels. dt-Tomato Red stably expressed MDA-MB-231 cells (target cells) were mixed with CFSE labelled MCF7 cells (non-target cells) at a ratio of 1:1, and then cocultured for 18 h with either control or c-Met specific chimeric antigen receptor T cells at a ratio of 1:2 separately. Cells were harvested and analyzed by flow cytometry. The data are presented as the mean ± SEM (error bar) from three replicates. *P< 0.05.

Given that UBR5 regulates IFN-γ-induced PD-L1 expression and that *Ubr5^-/-^* tumor growth is arrested from Day 10 onward 21, it is important to determine the role of PD-L1 in the impaired growth of *Ubr5^-/-^* tumors. We thus rescued PD-L1 in 4T1/*Ubr5^-/-^* cells with murine *Pdl1* to levels similar to those in WT 4T1 cells (**Figure 2A-C**) without affecting the expression of UBR5 (**Figure 2D**). Upon inoculation in mice, the m*Pdl1*-reconstituted 4T1/*Ubr5^-/-^* cells exhibited substantially enhanced growth compared with *Ubr5^-/-^* cells (**Figure 2E**). Analyses of tumor-infiltrating immune cells revealed that the number of CD8^+^ T cells and their cytolytic activity, manifested as increased granzyme B expression and decreased PD-1 expression, was strongly increased in *Ubr5^-/-^* tumors, which were subsequently reversed following the rescue of *Pdl1* or *Ubr5* expression (**Figure S2**,** Figure 2F**). The restoration also effected IFN-γ production of these CD8^+^ T cells (**Figure S3**), as well as the presence of CD25^+^ and FoxP3^+^ regulatory T cells (**Figure S2**,** Figure 2F**). Interestingly, the increased CD11c^+^ MHC Ⅱ^+^ mature dendritic cells (DCs) observed previously in mice bearing 4T1/*Ubr5*^-/-^ tumor 21, was also lost when either m*Pdl1* or h*UBR5* was reconstituted (**Figure S2, Figure 2F**). Accordingly, a significantly poorer prognosis was observed in the m*Pdl1*-reconstituted 4T1/*Ubr5^-/-^* group than that of the *Ubr5^-/-^* group (**Figure 2G**). Given that the UBR5 deficiency decreased PD-L1 levels in MDA-MB-231 cells (**Figure 1D**-**F**), we tested the idea if this could enhance the function of effector T cells, using the c-Met specific human chimeric antigen receptor (CAR) T cells on the human TNBC cells. We found that, indeed, UBR5 deficiency in the target cells rendered them more susceptible to CAR T-mediated killing (**Figure 2H**, **Figure S4**).

### Combined genetic targeting of *Ubr5* and *Pdl1* yields synergistic long term therapeutic effects

**Figure 3:**
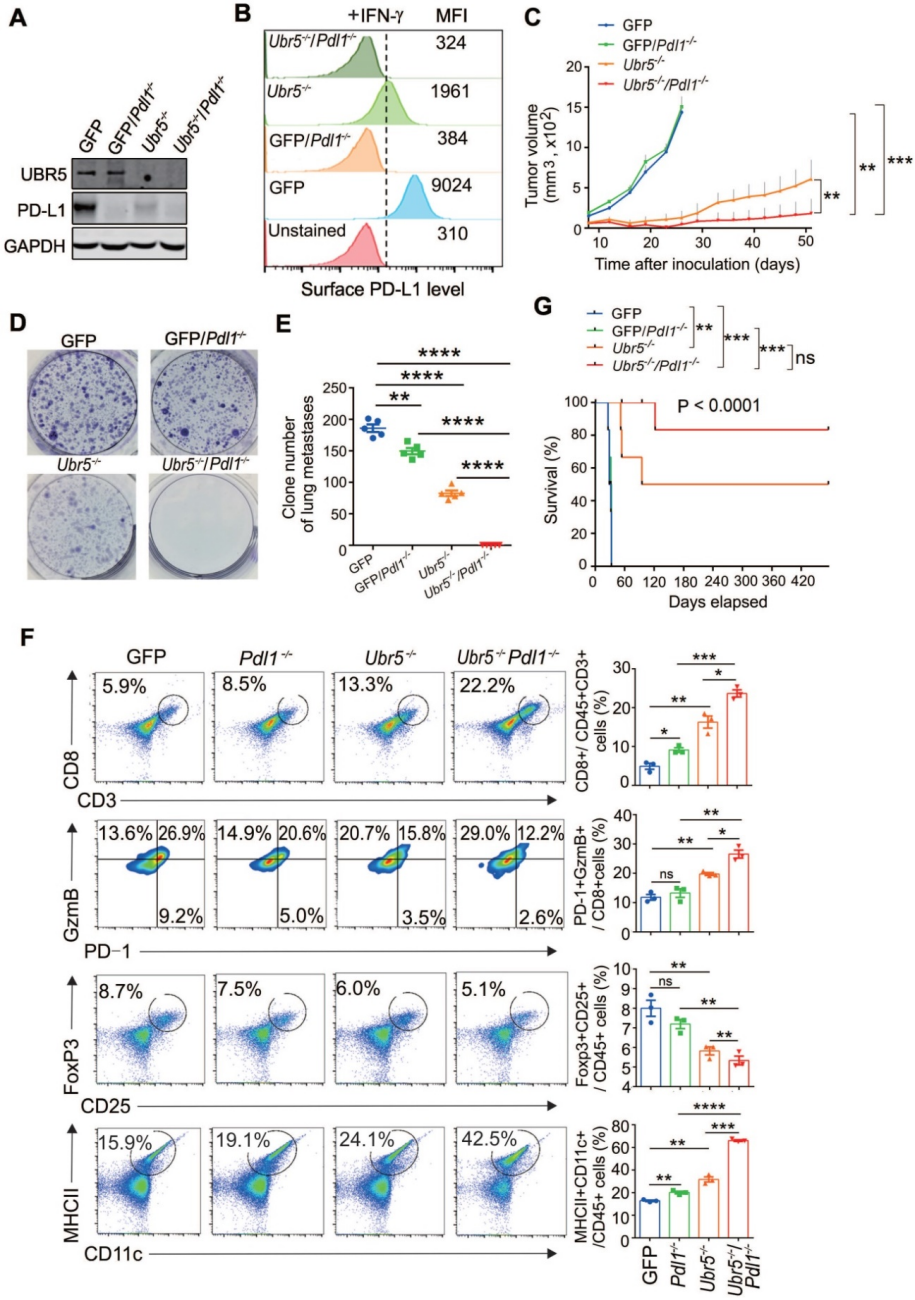
** Combined genetic targeting of *Ubr5* and *Pdl1* yields synergistic therapeutic effects.** (A-B) 4T1/GFP/*Pdl1^-/-^* and 4T1/*Ubr5^-/-^Pdl1^-/-^* cells were generated by CRISPR/Cas9 editing. WT, *Ubr5^-/-^*, GFP/*Pdl1^-/-^* or *Ubr5^-/-^Pdl1^-/-^* 4T1 cells were treated with 10 ng/mL IFN-γ for 24 h, and then the UBR5 and PD-L1 protein levels were detected by western blot (A) and surface PD-L1 levels were measured by flow cytometry analysis (B). (C) A total of 1×10^6^ WT, *Ubr5^-/-^*, GFP/*Pdl1^-/-^* or *Ubr5^-/-^Pdl1^-/-^* 4T1 cells were subcutaneously injected into the mammary pads to monitor tumor growth in WT (BALB/c) mice (n=6 mice per group). Tumor size was measured every 3 days. (D-G) WT, *Ubr5^-/-^*, GFP/*Pdl1^-/-^* or *Ubr5^-/-^Pdl1^-/-^* 4T1 cells (5×10^5^) were injected via the tail vein into 7-week-old female BALB/c mice (n=5 mice per group). Twelve days later, the mice were sacrificed, and single-cell suspensions were obtained from lung tissue to perform clonogenic assays in order to evaluate lung metastasis. Images of 6-well plates in a representative experiment are shown in (D), and colonies were quantified (E). (F) On Day 10 after tumor cells inoculation, CD8^+^ T cells infiltration and GzmB^+^/PD-1^-^/CD8^+^ T cells in tumor tissue from mice bearing 4T1 WT, *Ubr5^-/-^*, h*UBR5-*or m*Pdl1*-reconstituted *Ubr5^-/-^* tumor were analyzed by flow cytometry. Flow cytometry analyzed CD25^+^, Foxp3^+^ Tregs and CD11c^+^, MHC Ⅱ^+^ DCs in tumor-draining lymph nodes. n=3 mice per group. The survival curve is shown in (G). The results are presented as the mean ± SEM. ns, no significance, *P < 0.05, **P < 0.01, ***P < 0.001, ****P < 0.0001.

Next, we investigated whether combined abrogation of intrinsic UBR5 and PD-L1 expression has synergistic therapeutic benefits. GFP*/Pdl1^-/-^* and* Ubr5^-/-^Pdl1^-/-^* cell lines derived from the GFP and *Ubr5^-/-^* 4T1 cell lines were generated by CRISPR/Cas9 editing (**Figure 3A**-**B**). The cells were inoculated into the mammary pad of mice and the tumor growth was monitored over time (**Figure 3C**). The tumor growth of the *Ubr5^-/-^* group was dramatically reduced within 30 days, as we have reported previously 21, but gradually increased beyond 30 days. Tumor growth was considerably more arrested in the *Ubr5^-/-^Pdl1^-/-^* group compared with the other groups. Furthermore, the tumor did not recur in 83% of mice (4/6) in the *Ubr5^-/-^Pdl1^-/-^* group until Day 122. Markedly, no tumor recurrence was observed in these mice for more than 1 year. In contrast, no therapeutic benefit was observed in the GFP/*Pdl1^-/-^* group, which is consistent with the TNBC “cold tumor” theory 30.

To evaluate the effect of dual targeting UBR5 and PD-L1 on the spontaneous lung metastasis of 4T1 tumors, 5×10^5^ tumor cells were administrated i.v. to mice and lung metastasis was measured at Day 12 post injection using the 6-thioguanine clonogenicity assay (**Figure 3D**). We observed that the number of lung-colonizing *Ubr5^-/-^Pdl1^-/-^* tumor cells was significantly less than the number of colonizing *Ubr5^-/-^* tumor cells (**Figure 3E**). Notably, ~2 folds higher CD8^+^ T cell infiltration was observed in *Ubr5^-/-^Pdl1^-/-^* tumors than in *Ubr5^-/-^* tumors (**Figure 3F**). These infiltrating T cells were more active shown by an increased GzmB^+^/PD-1^-^ effector T population and decreased regulatory T cells (**Figure 3F**) and more GzmB and IFN-γ production (**Figure S5**). Increased production of IFN-γ by CD4^+^ T cells were also observed in the tumors of mice bearing 4T1/*Ubr5^-/-^Pdl1^-/-^* tumor (**Figure S5**). Furthermore, CD11c^+^ MHC Ⅱ^+^ mature DCs in 4T1/*Ubr5*^-/-^*Pdl1^-/-^* tumor significantly increased (**Figure 3F**). Consequently, the *Ubr5^-/-^Pdl1^-/-^* bearing mice exhibited prolonged survival for up to 420 days (**Figure 3G**). These results demonstrate that simultaneous blockade of UBR5 and PD-L1 expression has synergistic therapeutic benefits with a strong impact on the infiltrating immune cells.

### UBR5 globally regulates IFN-γ-mediated pathways and stimulated genes

**Figure 4:**
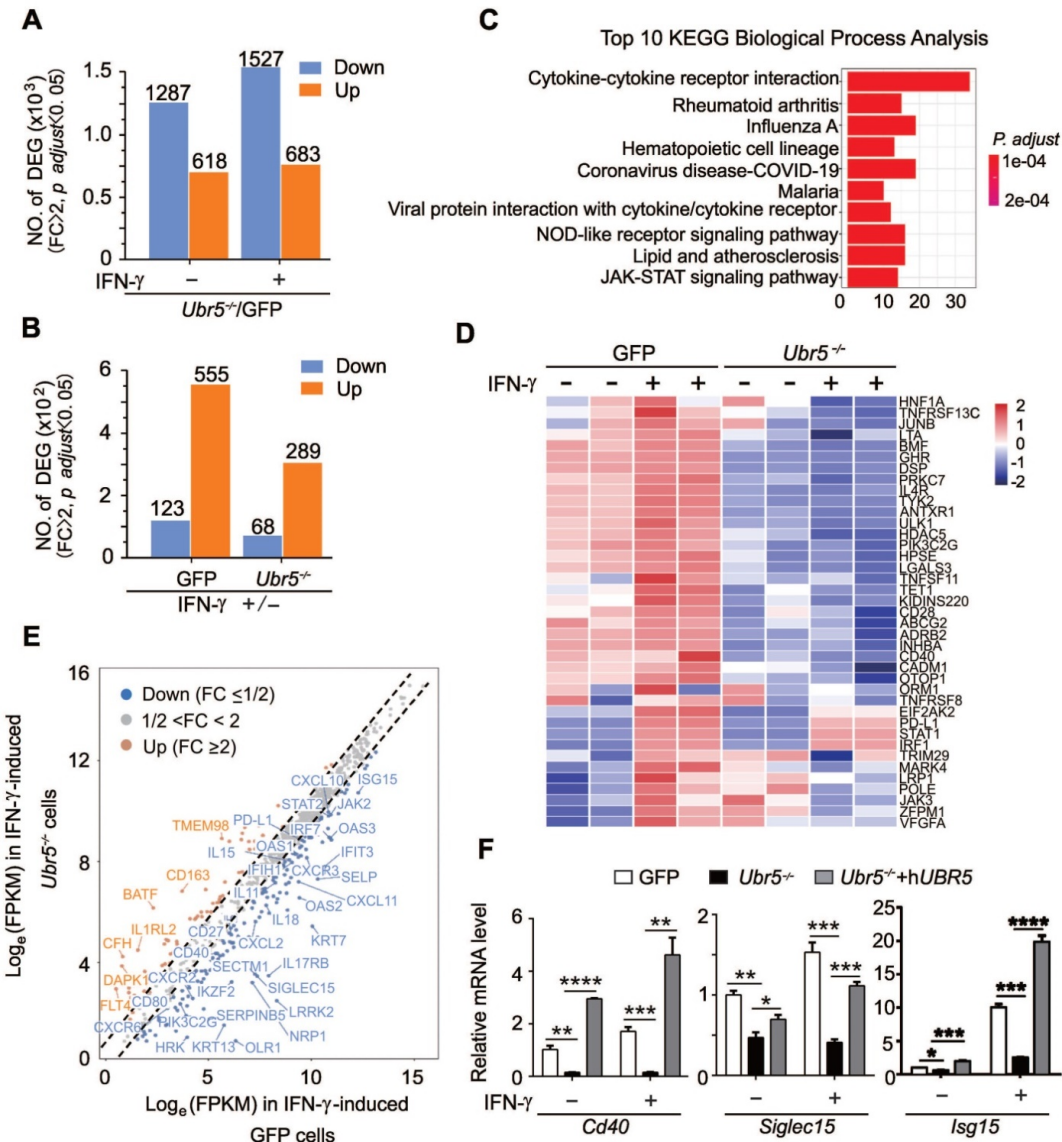
** UBR5 globally regulates IFN-γ-mediated pathways and stimulated genes.** (A) Numbers of differentially expressed genes (DEGs) in *Ubr5^-/-^* compared with GFP 4T1 cells with or without IFN-γ treatment. Fold change (FC) ≥ 2, p. adjust ˂ 0.05. (B) Numbers of DEGs in GFP 4T1 cells or *Ubr5^-/-^* 4T1 cells with IFN-γ treatment compared with that without IFN-γ treatment. Fold change (FC) ≥ 2, p. adjust ˂ 0.05. (C) Kyoto Encyclopedia of Genes and Genomes (KEGG) pathway enrichment of the top 10 biological process between IFN-γ-treated GFP and *Ubr5^-/-^* 4T1 cells. (D) Heatmap depicting the mRNA levels of selected ISGs in GFP and *Ubr5^-/-^* 4T1 cells with or without 10 ng/mL IFN-γ induction for 24 h. (E) Scatterplot diagram of genes responding to IFN-γ differently in *Ubr5^-/-^* cells compared to that in GFP cells. The grey dots represent unchanged expression (1/2<FC<2) in both cell lines. Upregulated ISGs with fold change (FC) values >2 in* Ubr5^-/-^* cells compared with GFP cells are shown with orange dots, while downregulated ISGs with fold change (FC) values <1/2 are shown with blue dots. (F) The mRNA levels of* Cd40*, *Siglec15* and *Isg15* were examined by qPCR analysis in WT, *Ubr5^-/-^* and h*UBR5*-reconstituted *Ubr5^-/-^* 4T1 cells treated with or without IFN-γ for 24 h. All experiments were repeated three times, and the data are presented as the mean ± SEM. *P < 0.05, **P < 0.01, ***P < 0.001, ****P < 0.0001.

Since UBR5 could enhance IFN-γ-induced PD-L1 expression to promote tumor growth, it was of interest to explore whether there are other IFN-γ responsive factors affected by UBR5, we performed transcriptome profiling with RNA-sequencing (RNA-seq) in 4T1/GFP and 4T1/*Ubr5^-/-^* cells treated with or without IFN-γ. The RNA-seq data showed that there were more genes downregulated than upregulated in *Ubr5^-/-^* cells compared with GFP 4T1 cells no matter with or without IFN-γ stimulation (**Figure 4A**). A total of 555 genes were induced with IFN-γ in 4T1/GFP cells, while significantly fewer (289 genes) were induced in 4T1/*Ubr5^-/-^* cells (**Figure 4B**). In addition, more genes were downregulated in 4T1/GFP cells (123 genes in total) than in 4T1/*Ubr5^-/-^* cells (68 genes in total) (**Figure 4B**). RNA-seq data of IFN-γ-treated WT and *Ubr5^-/-^* 4T1 cells were further evaluated both by Kyoto Encyclopedia of Genes and Genomes (KEGG) pathway enrichment and Gene Ontology (GO) functional annotation analysis. KEGG pathway enrichment analysis revealed that UBR5 was highly correlated with multiple signaling pathways including the JAK-STAT pathway and cytokine-receptor interactions, which suggests that UBR5 is involved in relatively broad regulation of the IFN-γ stimulation pathway (**Figure 4C**). The pathways of positive response to external stimulus and regulation of T cell activation/leukocyte proliferation ranked in the top 20 GO biological processes in the enrichment analysis (**Figure S6**). Interestingly, in selected interferon-stimulated gene (ISG) subsets, a set of genes respond to IFN-γ in GFP cells but completely not or only mildly responsive in *Ubr5^-/-^* cells (**Figure 4D**). Genes responding to IFN-γ differently in 4T1/*Ubr5^-/-^* cells, including the immune costimulatory and checkpoint genes *Cd40* 31 and* Siglec15* 32 significantly decreased in IFN-γ-induced 4T1/*Ubr5^-/-^* cells compared with IFN-γ-induced 4T1/GFP cells (**Figure 4E**), while expression of ISG resistance signature (ISG.RS) genes, such as ISG* Isg15* 33, *JAK2*, *OAS3*, *OAS1*, *IRF7*, *OAS2*, *IFIT3* and *IFIH1* decreased (**Figure 4E**). These genes are predominantly expressed in cancer cells, albeit with variable expression 34. Further analysis utilizing WT, *Ubr5^-/-^* and h*UBR5*-reconstituted *Ubr5^-/-^* 4T1 cells confirmed that the transcription of the *Cd40*,* Siglec15* and *Isg15* genes was affected by the expression of UBR5 (**Figure 4F**).

### UBR5 is crucial for IFN-γ-mediated activation of *STAT1* and *IRF1* transcription

**Figure 5:**
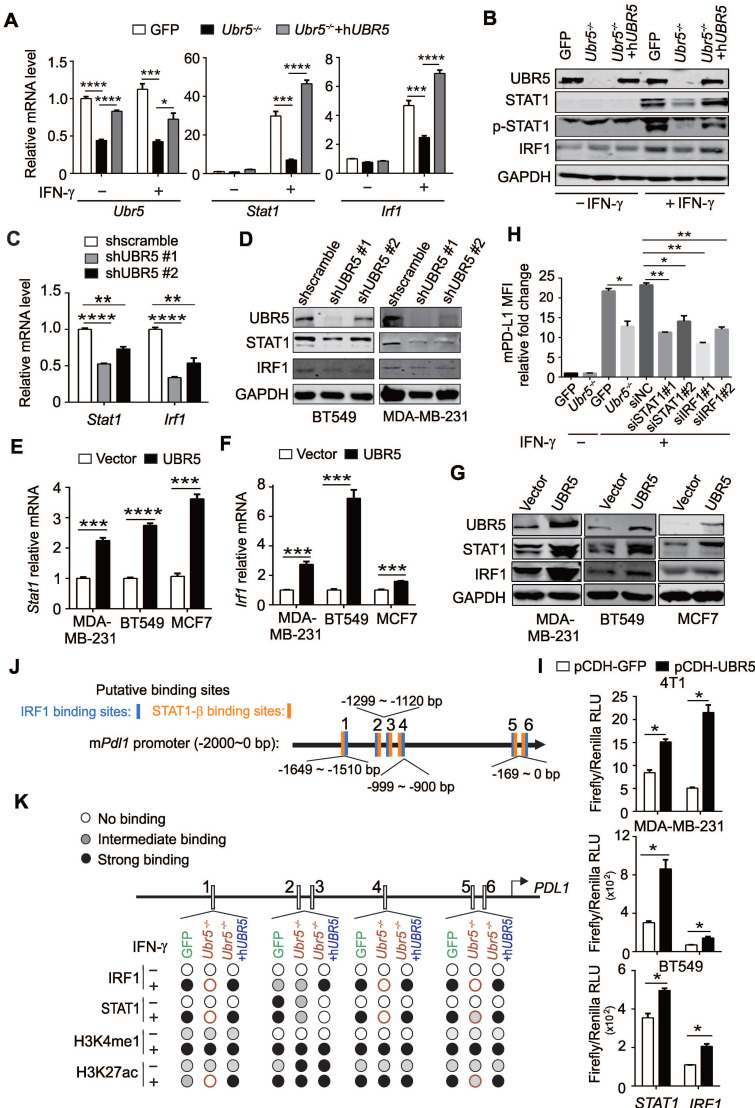
** UBR5 is crucial for IFN-γ-induced activation of *STAT1* and *IRF1* transcription.** (A-B) The mRNA (A) and protein (B) levels of UBR5, STAT1, pSTAT1 and IRF1 were detected in WT, *Ubr5^-/-^* and h*UBR5*-reconstituted *Ubr5^-/-^* 4T1 cells with or without IFN-γ stimulation. (C-D) The mRNA (C) and protein levels (D) of STAT1 and IRF1were detected in IFN-γ-treated UBR5-knockdown BT549 (C and D) and MDA-MB-231 (D) stable cell lines. (E-G) BT549, MDA-MB-231 and MCF7 cells were transfected with either an empty vector or UBR5 plasmids. 24 hours later, the cells were treated with IFN-γ for 24 h. Then the mRNA and protein levels of STAT1 (E and G) and IRF1 (F and G) were measured by qPCR and western blot, respectively. (H) Surface PD-L1 levels were detected in IFN-γ-treated GFP, *Ubr5^-/-^* 4T1 cells and GFP 4T1 cells treated with siNC, siSTAT1 or siIRF1. GAPDH was used for normalization. (I) Luciferase reporter vectors containing either *STAT1* or *IRF1* promoter regions were cotransfected with an empty vector or UBR5 plasmids into the indicated cells. After 24 h, the transfected cells were treated with IFN-γ stimulation for 24 h, the cells were lysed to perform luciferase assay. The results are presented as the mean ± SEM from three individual experiments. *P < 0.05, **P < 0.01, ***P< 0.001, ****P < 0.0001. (J) The IRF1 and STAT1 binding sites in the m*Pdl1* promoter region were predicted using the ALGGEN website. (K) Summary of the results of a ChIP assay using anti-IRF1, STAT1, H3K4me1 and H3K27ac antibodies in WT, *Ubr5^-/-^* or h*UBR5*-reconstituted *Ubr5^-/-^* 4T1 cells after treatment with or without IFN-γ.

IFN-γ is generally considered the most prominent soluble inducer of PD-L1 and the JAK1/2-STAT1/3-IRF1 signaling axis has been shown to play a central role in the IFN-γ-mediated induction of PD-L1 11, 35. RNA-seq-based transcriptomic analyses suggested that the JAK-STAT signaling pathway (**Figure 4C**) played a role in the regulation of IFN-γ-stimulated PD-L1 by UBR5. The expression levels of JAK3, STAT2 and IRF7 were also significantly different between IFN-γ-treated GFP and *Ubr5^-/-^* 4T1 cells (**Figure 4E**). We then evaluated PD-L1-targeting transcription factors including JAK1, JAK2, JAK3, STAT1, STAT2, STAT3, IRF1, IRF7, and TYK2 (**Figure S7**), and found that the IFN-γ-stimulated mRNA and protein levels of IRF1 and STAT1 were decreased in UBR5-silenced 4T1 (**Figure 5A-B**), BT549 (**Figure 5C-D**) and MDA-MB-231 (**Figure 5D**) cells. Reciprocally, overexpression of UBR5 increased the mRNA (**Figure 5E- F**) and protein levels (**Figure 5G**) of *STAT1* and *IRF1* in different human breast cancer cell lines. The protein levels of pSTAT1 increased in UBR5 overexpression BT549 cells compared to the control cells (**Figure S8**). The phosphorylation level of STAT1 also markedly decreased in 4T1/*Ubr5^-/-^* cells (**Figure 5B**). Consistently, both the mRNA (**Figure 5A**) and protein (**Figure 5B**) levels of STAT1 and IRF1, as well as the levels of pSTAT1 were restored after h*UBR5* was reconstituted in 4T1/*Ubr5^-/-^* cells. The mRNA levels of *Jak1*,* Jak2*,* Stat3*,* Tyk2* altered in a similar pattern as those of *Stat1* and *Irf1* in GFP, *Ubr5^-/-^* and h*UBR5*-reconstituted *Ubr5^-/-^* 4T1 cells; however, *Jak1* gene did not respond to IFN-γ treatment, and the mRNA levels of *Jak2*, *Stat3* and *Tyk2* increased less than 2 fold in IFN-γ-induced 4T1/GFP cells, which is inconsistent with *Pdl1* mRNA level changes in 4T1 cells with or without IFN-γ stimulation (**Figure S7**). To further evaluate the roles of these genes in IFN-γ-induced *PDL1* transcription in 4T1 cells, JAK3, STAT1, STAT2, IRF1, and IRF7 were silenced individually through siRNA (**Figure S9**). The expression of *STAT1* and *IRF1* transcripts, but not *JAK3*, *STAT2* or *IRF7* transcripts were required for IFN-γ-induced *PDL1* transcription (**Figure 5H, S9**). These results suggest that upon IFN-γ-activation, UBR5 enhanced *PDL1* transcription is mediated through *STAT1* and *IRF1*.

In eukaryotic cells, mRNA homeostasis is achieved through a balance between mRNA synthesis and degradation. Therefore, we sought to investigate whether UBR5 affects *STAT1* and *IRF1* mRNA by affecting its stability or transcription. 4T1 cells were treated with the transcriptional inhibitor actinomycin D and the mRNA levels of *STAT1* and *IRF1* were evaluated over time. The half-lives of both *STAT1* and *IRF1* mRNA showed no differences in WT, *Ubr5^-/-^* and h*UBR5*-reconstituted *Ubr5^-/-^* 4T1 cells (**Figure S10**). Next, we explored whether UBR5 affects the transcription of *STAT1* and *IRF1* via dual-luciferase reporter assay. Human *STAT1* (from -972 to +884) and *IRF1* (from -820 to +138) promoters and mouse *Stat1* (from -2000 to +100) and *Irf1* (from -2000 to +100) promoters were cloned into pGL3 plasmids separately. These reporter plasmids were transiently cotransfected with pCDH-UBR5/pCDH-eGFP plasmids into MDA-MB-231, BT549 or 4T1 cell lines separately to detect *STAT1* and *IRF1* transcription activity. The results showed that UBR5 indeed enhanced the transcriptional activity of the *STAT1* and *IRF1* promoters in BT549, MDA-MB-231 and 4T1 cells (**Figure 5I**). These data confirm that UBR5 plays an essential role in IFN-γ-induced activation of *STAT1* and *IRF1* transcription by enhancing synthesis rather than slowing degradation.

To further explore whether the effect of UBR5 on the transcription of *PDL1* is mediated by the binding of STAT1-IRF1 in the *PDL1* promoter region, we carried out chromatin immunoprecipitation (ChIP)-qPCR assays to analyse the enrichment of *Stat1* and *Irf1* in the m*Pdl1* promoter region. Six putative *Stat1* binding sites and six putative *Irf1* binding sites were predicted by the ALGGEN website (**Figure 5J**). Enhanced enrichment of *Stat1* and *Irf1* (in predicted binding sites 1 and 4 and predicted binding sites 5 and 6) in the m*Pdl1* promoter was observed in WT and h*UBR5*-reconstituted *Ubr5^-/-^* 4T1 cells compared to *Ubr5^-/-^* 4T1 cells, which further supported the idea that the UBR5-mediated enhancement of IFN-γ-induced *PDL1* transcription is dependent on STAT1 and IRF1 (**Figure 5K**, **Figure S11**).

Increased post-translational histone modification, such as H3K4me1 and H3K27ac, have been reported to be responsible for the overexpression of PD-L1 as well as immune evasion in cancer 36, 37. Although the status of H3K4me1 and H3K27ac differed when cells were stimulated with IFN-γ at sites 1, 2, 3, 4, 5, and 6, there were no differences among the 4T1 WT, *Ubr5^-/-^* and h*UBR5*-reconstituted *Ubr5^-/-^* cell lines (**Figure S12**). In addition, treatment with trichostatin A (TSA), a histone deacetylase (HDAC) inhibitor, and 5-aza-2'-deoxycytidine (5'-AZA-dC), a DNA methylation inhibitor did not change the expression of PD-L1 in 4T1 cell surface regardless of whether UBR5 was expressed (**Figure S13**). These results suggest that histone modifications such as H3K4me1 and H3K27ac are not involved in the regulation of PD-L1 expression by UBR5.

### UBR5-mediated transactivation of *PDL1* is independent of the E3 ligase activity

**Figure 6:**
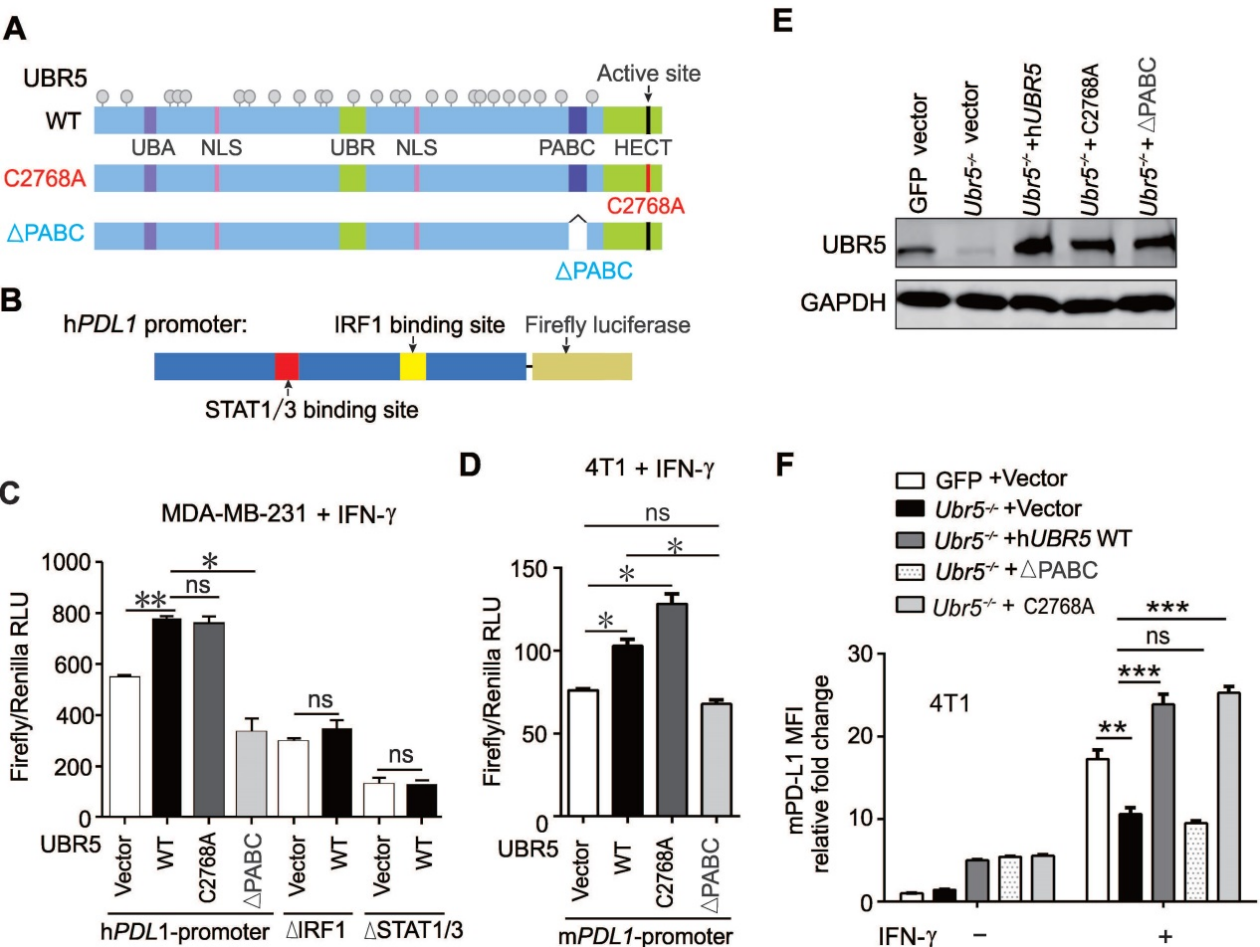
** UBR5-mediated transactivation of *PDL1* is independent of its E3 ubiquitin ligase.** (A-D) MDA-MB-231 cells were cotransfected with WT, HECT-mutation (UBR5-C2768A) or PABC-deletion UBR5 (A) together with luciferase reporters containing the human* PDL1* WT promoter or a promoter with 2 deletions, including the STAT1/3 or IRF1 binding sites (B), and then stimulated with IFN-γ for 24 h. Luciferase activity was measured in cell lysates of MDA-MB-231 (C) and 4T1 cells (D) by dual luciferase assay. (E-F) The protein levels of UBR5 (E) and PD-L1 (F) were measured in IFN-γ-treated GFP, *Ubr5^-/-^*, h*UBR5*-reconstituted *Ubr5^-/-^* 4T1 cells and *Ubr5^-/-^* 4T1 cells reconstituted with either h*UBR5*-C2768 or h*UBR5*-ΔPABC. The results are presented as the mean ± SEM from three individual experiments. ns, no significance, *P < 0.05, **P < 0.01, ***P < 0.001.

Given that UBR5 was found to be responsible for IFN-γ-induced *STAT1* and* IRF1* transcription, we further explored that which domain of UBR5 (**Figure 6A**) is critical to its regulation. The human *PDL1* promoter was cloned and inserted it into a pGL3-based firefly luciferase reporter vector (**Figure 6B**), and transiently cotransfected the vector with plasmids encoding wild type UBR5, the HECT domain mutant C2768A or the PABC domain deletion (∆PABC) of UBR5 into MDA-MB-231 cells. The results showed that UBR5 increased the transcriptional activity of the *PDL1* promoter (**Figure 6C**). The UBR5-ΔPABC mutant completely lost the IFN-γ-inducibility on the *PDL1* promoter, suggesting that this domain is pivotal for regulation. *PDL1* promoter constructs with site-directed mutations in the binding sites of IRF1 or STAT1/3 11 were generated (**Figure 6B**), and transiently cotransfected into MDA-MB-231 cells with expression vector of UBR5 and the variants. Luciferase activity data indicated that the IRF1 and STAT1/3 binding sites were essential for the transcriptional activity of the *PDL1* promoter by UBR5 upon IFN-γ stimulation (**Figure 6C**). Consistently, a highly analogous response was observed in IFN-γ-stimulated 4T1 cells on the mouse *Pdl1* promoter (**Figure 6D**).

To further confirm that PABC is required for IFN-γ-induced *PDL1* transcription, we transiently transfected plasmids encoding h*UBR5*, h*UBR5*-ΔPABC or h*UBR5*-C2768A respectively into 4T1/*Ubr5^-/-^* cells (**Figure 6E**) and performed flow cytometry assays to detect PD-L1 surface protein levels. Consistent with the dual-luciferase reporter assay results, the PABC domain, but not the E3 ligase catalytic site, was essential for the regulation of IFN-γ-induced PD-L1 expression (**Figure 6F**). Additionally, we corroborated the dispensability of UBR5 ubiquitin ligase activity by treating BC cells with MG132, an inhibitor of the 26S proteasome. The reduced surface PD-L1 level observed in UBR5-depleted cells did not result from increased protein degradation through the proteasome (**Figure S14**). Taken together, UBR5 promotes PD-L1 in a manner dependent on its PABC domain and independent of the E3 ligase activity.

### UBR5 transactivation of *STAT1* and *PDL1* is mediated by PKR

**Figure 7:**
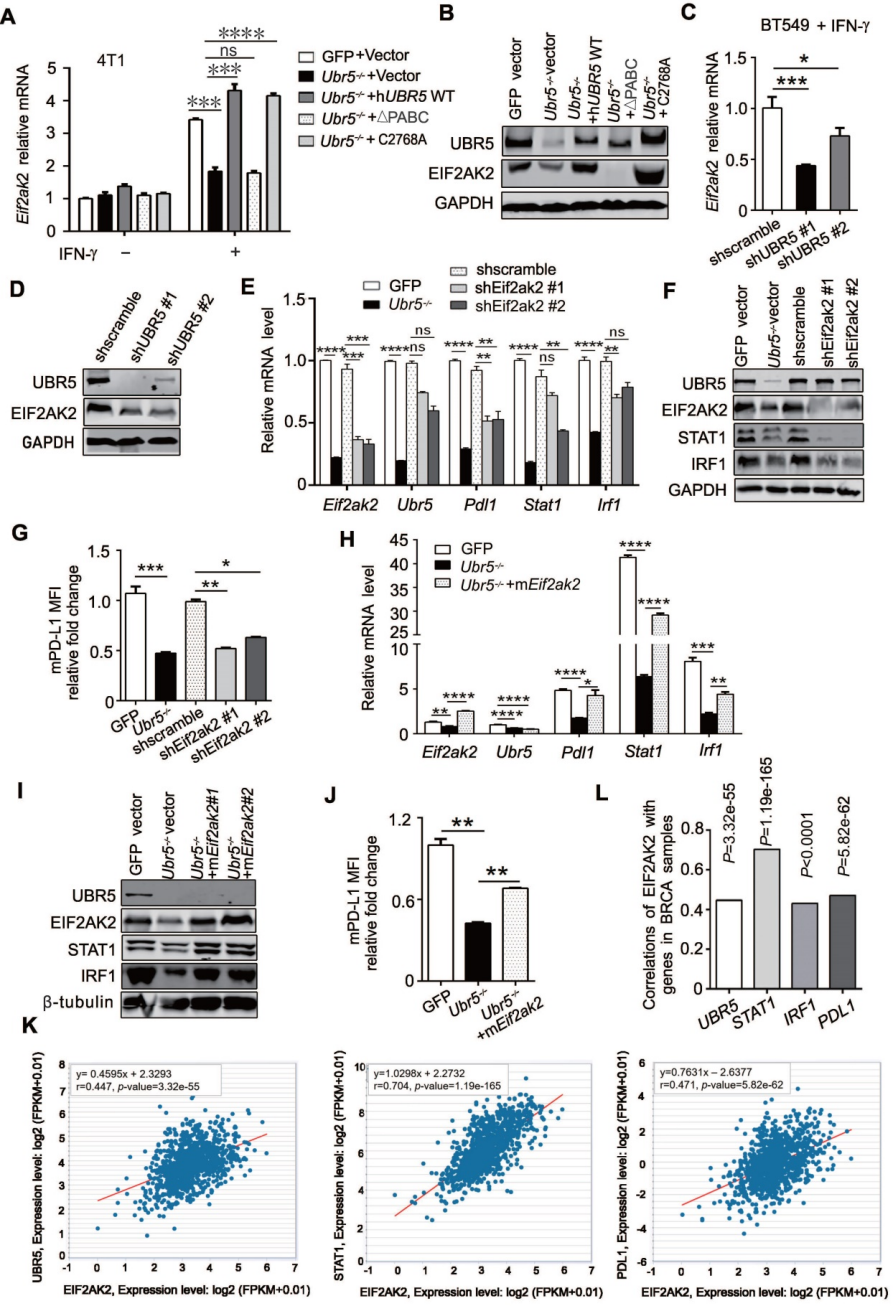
** The transactivation of UBR5 to *STAT1* and *PDL1* is mediated by protein kinase RNA-activated.** (A-B) The mRNA (A) and protein levels (B) of EIF2AK2 in WT, *Ubr5^-/-^*, h*UBR5*-reconstituted *Ubr5^-/-^* 4T1 cells and *Ubr5^-/-^* 4T1 cells reconstituted with either h*UBR5*-C2768A or h*UBR5*-ΔPABC were measured by qPCR and western blot analysis after cells were treated with or without IFN-γ. (C-D) The mRNA (C) and protein levels (D) levels of EIF2AK2 in WT and UBR5-knockdown BT549 cells were measured by qPCR and western blot analysis after cells were treated with IFN-γ for 24 h. (E-G) The mRNA (E) and protein levels of UBR5, STAT1, IRF1 and EIF2AK2 (F) and relative fold changes of cell-surface PD-L1 levels (G) in IFN-γ-treated WT, *Ubr5^-/-^*, and 4T1 cells with stable knockdown by *shEif2ak2* #1 and *shEif2ak2* #1. Shscramble served as the control. (H-J) The mRNA levels (H) of *Eif2ak2*, *Ubr5*, *Pdl1*, *Stat1* and *Irf1*, the protein levels of UBR5, EIF2AK2, STAT1 and IRF1 (I) and the surface levels of PD-L1 (J) were measured in IFN-γ-treated 4T1 WT, *Ubr5^-/-^* and m*Eif2ak2*-reconstituted *Ubr5^-/-^* cell lines. The results are presented as the mean ± SEM from three individual experiments. ns, no significance, *P< 0.05, **P < 0.01, ***P< 0.001, ****P < 0.0001. (K) The correlations of *EIF2AK2* with *UBR5*, *PDL1* and *STAT1* mRNA levels were assessed in the TCGA BRCA database (1104 samples) by starBase database. (L) The summary correlations of *EIF2AK2* with *UBR5*, *PDL1*, *STAT1* and *IRF1* mRNA levels (normalizated to GAPDH) were analysed in the TCGA BRCA database by starBase and GEPIA database respectively.

Through RNA-seq **(Figure 4D)** and previous mass spectrometry (MS) analysis 29, we observed a significant downregulation of *EIF2AK2* in RNA and protein levels, the gene that encodes protein kinase RNA-activated (PKR), in *Ubr5^-/-^* cells compared with WT 4T1 cells 29. PKR can be activated by IFN-γ mRNA 38 and plays an important role in interferon and dsRNA‐signaling pathways by modulating the transcriptional function of STAT1 39. In addition, PKR can indirectly activate the modification of IRF1 and activation of its DNA-binding activity in a protein kinase-dependent manner 40. Therefore, we speculated that UBR5 might regulate STAT1 and IRF1 through PKR. To test this possibility, we first evaluated *Eif2ak2* mRNA levels in 4T1 WT, *Ubr5^-/-^*, h*UBR5*-reconstituted, h*UBR5*-ΔPABC and h*UBR5*-C2768A-reconstituted *Ubr5^-/-^* cells with or without IFN-γ treatment. Interestingly, the alterations in* Eif2ak2* mRNA (**Figure 7A**) and protein (**Figure 7B**) levels were consistent with the patterns of *Stat1*, *Irf1* and *Pdl1* alterations with IFN-γ induction when the expression of UBR5 changed. Consistently, the *Eif2ak2* mRNA (**Figure 7C**) and protein (**Figure 7D**) levels were decreased in BT549 cells after UBR5 was knocked down. The EIF2AK2 was expressed in response to IFN-γ in h*UBR5*-C2768A-reconstituted *Ubr5^-/-^* cells but not in h*UBR5*-ΔPABC-reconstituted *Ubr5^-/-^* cells (**Figure 7A**-**B**), which was consistent with our previous observation in **Figure 6C-F** that PD-L1 regulation by UBR5 is dependent on the PABC domain.

To further confirm that EIF2AK2 affects the mRNA levels of *STAT1* and *IRF1*, we generated *Eif2ak2*-knockdown 4T1 cells via shRNA (**Figure 7E**). The mRNA levels of *Pdl1*, *Stat1* and* Irf1* (**Figure 7E**) and the protein levels of STAT1, IRF1 (**Figure 7F**) and PD-L1 (**Figure 7G**) decreased in 4T1 cells after *Eif2ak2* knockdown. Conversely, the mRNA and protein levels of STAT1, IRF1 and PD-L1 were restored after we reconstituted m*Eif2ak2* expression in *Ubr5^-/-^* cells compared with GFP and *Ubr5^-/-^* 4T1 cells (**Figure 7H-J**). However, compared with WT cells, the cell surface protein level of PD-L1 restoration was not completely rescued in m*Eif2ak2*-reconstituted cells, suggesting that additional mechanisms might be involved in the regulation of UBR5-mediated PD-L1 surface upregulation other than PKR*.* These data suggest that the transactivation of *STAT1* and *PDL1* by UBR5 is mediated by PKR.

To investigate whether our observations match the scenario in clinical human breast cancer samples, we performed bioinformatic analysis with data from TCGA breast cancer invasive carcinoma (BRCA) RNA-seq database by GEPIA and starBase database respectively. Moderate and above correlations between *EIF2AK2* and *UBR5*, *STAT1*, *IRF1* or *PDL1* expression (Pearson correlation: R=0.447, R=0.704, R=0.43 and R=0.471, respectively) were observed (**Figure 7K**-**L**), which supports the idea that *EIF2AK2* expression is highly positively correlated with those of *UBR5*, *STAT1*,* IRF1* and *PDL1*.

PKR can mediate the activation of IFN-γ-stimulated STAT1 by regulating p38 MAPK and is required for efficient activation of JNK by IFN-γ 41. To further explore whether UBR5 activates PKR-mediated STAT1 and PD-L1 through p38 or JNK, we used the PKR inhibitor oxindole/imidazole compound (C16) to treat GFP and *Ubr5^-/-^* 4T1 cells with or without IFN-γ stimulation. The results showed that there were no differences of the protein levels of STAT1, p38 and JNK (and their phosphorylated forms) with or without PKR inhibition in GFP and *Ubr5^-/-^* 4T1 cells in the presence of IFN-γ (**Figure S15**). Also, the surface levels of PD-L1 protein were not different between GFP cells and* Ubr5^-/-^* 4T1 cells treated with C16 (**Figure S16**), suggesting that PKR is involved in UBR5-mediated activation of *STAT1* and *PDL1* transcription in a kinase-independent manner. The kinase activity independent PKR was also observed in PKR-activated NF-κB signaling pathway 42, 43, as well as in broad mechanisms of inflammasome-mediated caspase-1 activation 44.

Since the PABC domain is required for the regulation of EIF2AK2 by UBR5 (**Figure 7A**-**B**) and plays a role in enhancing mRNA stability 45, we further explored whether UBR5 controlled *Eif2ak2* by maintaining the latter's mRNA stability. IFN-γ-pretreated cells were treated with the transcriptional inhibitor actinomycin D for different times, and then the mRNA levels of *Eif2ak2* were evaluated. The half-lives of *Eif2ak2* mRNA were not different among 4T1 WT, *Ubr5^-/-^* and h*UBR5*-reconstituted cells (**Figure S17**). This result suggests that UBR5 regulates *Eif2ak2* not by enhancing its mRNA stability, but by increasing the transcription of *Eif2ak2*.

## Discussion

As PD-L1 is a ligand for the critical immune checkpoint molecule PD-1, the regulation of PD-L1 expression is complex and can occur at genetic, transcriptional and posttranscriptional levels. Here, we demonstrate that UBR5 is required for IFN-γ-induced PD-L1 expression in breast cancer, and that transactivation of *PDL1* gene expression by UBR5 is mediated through the PKR/STAT1/IRF1 pathway. Furthermore, we show that PD-L1 regulation by UBR5 is dependent on the PABC domain but not the E3 ligase activity of UBR5. More significantly, the augmented expression of PD-L1 by UBR5 can enhance the latter's function in promoting tumor growth and metastasis. Lastly, combined blockade of UBR5 and PD-L1 leads to a synergistic therapeutic benefit in eradicating tumor growth and prolong the survival or even to a potential cure (**Figure 3**), although we need to more rigorously corroborate the synergism by other ways of targeting of UBR5 and PD-L1 than the genetic means.

However, since the UBR5 protein does not contain a DNA binding motif, it is still unclear how UBR5 regulates *EIF2AK2*, although there is evidence that UBR5 can enhance its transcription through the UBR5 PABC domain. Previous studies have implied that UBR5 can regulate gene transcription through several mechanisms involving three different stages: initiation of RNA transcription by transcription factors and their associated co-activators 46, RNA elongation, and RNA processing and nuclear export 47. It seems from our observations that UBR5 does not affect *EIF2AK2* mRNA stability, thus UBR5 may promote *EIF2AK2* expression through other mechanisms at one or more of these three stages. UBR5 is overexpressed in many cancer types 20, 21, 48-54 and associated with poor prognosis in a variety of cancers 48-51, 55, 56. *EIF2AK2*-encoded PKR also plays a critical role in tumorigenesis. High PKR expression is linked with prognosis 57-62 and tumor malignancy in multiple cancer types 63-65. In addition, high PKR expression is related to genomic instability 66, reduced survival and shortened remission period 67. We showed that UBR5 could influence the transcription of *EIF2AK2*. Interestingly, we observed significant co-expression between *UBR5* and *EIF2AK2* in the TCGA mRNA database (**Figure 7K**). It's worth noting that PKR may also be upregulated by IFN-γ indirectly by UBR5-induced changes in the tumor microenvironment and it is of interest to further delineate the role UBR5 in the regulation of PKR expression *in vivo*.

We demonstrate that UBR5 is broadly and critically involved in the IFN-γ signaling pathway and in the regulation of immune checkpoints (**Figure 4**). Furthermore, our previous study 21 showed that IFN-γ mRNA levels were elevated in 4T1/*Ubr5^-/-^* tumors compared to WT. As a cytokine, IFN-γ plays a dual role in tumor immune response. On the one hand, IFN-γ inhibits the occurrence of tumor and promotes the apoptosis of tumor by regulating immune responses and cell cycle, promoting cell apoptosis, and inhibiting angiogenesis 68, 69. On the other hand, IFN-γ can promote immune escape of tumor cells by regulating the TME 70-79. However, there is little research on the conversional mechanisms of IFN-γ between the two contrasting activities, which also illustrates that IFN-γ has great prospects in the field of tumor immunotherapy.

It is noted that UBR5 can upregulate *PDL1* transcription to promote tumor immune evasion and that restoration of PD-L1 expression in UBR5-deficient tumor decreases T cell infiltration and restores malignancy, as was indirectly proven by the combination therapy targeting both UBR5 and PD-L1 in the TNBC mouse model. Therefore, dual targeting UBR5 and PD-L1 has better efficacy than single targeting for breast cancer, and potentially for other cancer types as well. These areas represent interesting directions for future work.

Together, our *in vivo* and *in vitro* results reveal for the first time, that UBR5 is a key player in the transcriptional regulation of IFN-γ-induced *PDL1* and *ISGs*, and in directing cancer immune evasion. These findings provide strong evidence and rationale for targeting both UBR5 and PD-L1 as a novel approach to enhance the efficacy of immune checkpoint blockade-based therapy against breast cancer, particularly TNBC.

## Supplementary Material

Supplementary figures and tables.

## Abbreviations

ChIP: Chromatin immunoprecipitation
DCs: Dendritic Cells
EGF: Epidermal growth factor
GO: Gene Ontology
GrzmB: Granzyme B
HECT: homologous to E6-AP C-terminus
IFN-γ: Interferon-γ
ILs: Interleukins
IRF: Interferon regulatory factor
ISGs: IFN-γ-stimulated genes
JAK: Janus kinase
MFI: Mean fluorescence intensity
NF-κB: Nuclear factor-κB
PABC: Poly adenylate binding C terminal
PD-L1: Programmed cell death-ligand1
PD-1: Programmed cell death protein 1
PKR: Protein kinase RNA-activated
PI3K: Phosphatidylinositol 3-kinase
STAT: Signal transducer and activator of transcription
TCGA: The cancer genome atlas
TME: Tumor microenvironment
TNBC: Triple negative breast cancer
TNF-α: Tumor necrosis factors α
UBR5: E3 component N-recognin 5
