# Supplementary material for: UBR5 promotes tumor immune evasion through enhancing IFN-γ-induced PDL1 transcription in triple negative breast cancer
Source: Theranostics. 2022 Jul 4;12(11):5086–102. doi: 10.7150/thno.74989 (PMC9274738; doi:10.7150/thno.74989)
Supplement: Supplementary file 1 — Supplementary figures and tables. [file thnov12p5086s1.pdf]

Supplementary Materials for

UBR5 promotes tumor immune evasion through enhancing IFN-γ-induced *PDL1* transcription in triple negative breast cancer

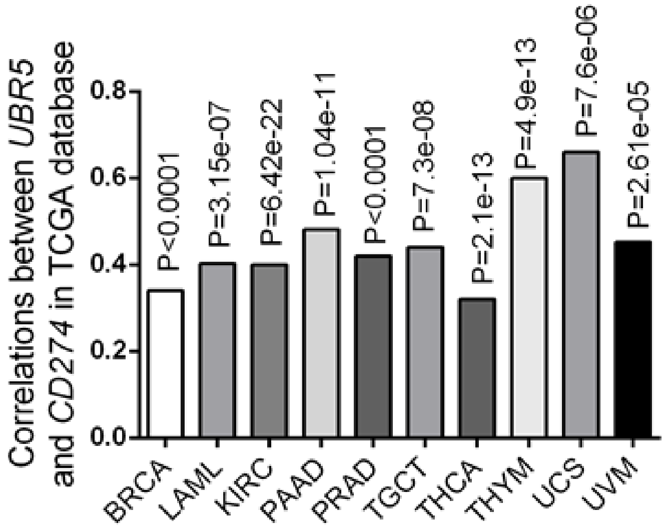

Figure S1. ***UBR5*** **and** ***PDL1*** **are positively correlated in multiple cancer types.**

The correlation of mRNA expression between *UBR5* and *PDL1* was assessed in TCGA database, normalized by GAPDH. Breast Invasive Carcinoma (BRCA), Acute Myeloid Leukemia (LAML), Kidney Renal Clear Cell Carcinoma (KIRC), Pancreatic Adenocarcinoma (PAAD), Prostate Adenocarcinoma (PRAD), Testicular Germ Cell Tumors (TGCT), Thyroid Carcinoma (THCA), Thymoma (THYM), Uterine Carcinosarcoma (UCS) and Uveal Melanoma (UVM)

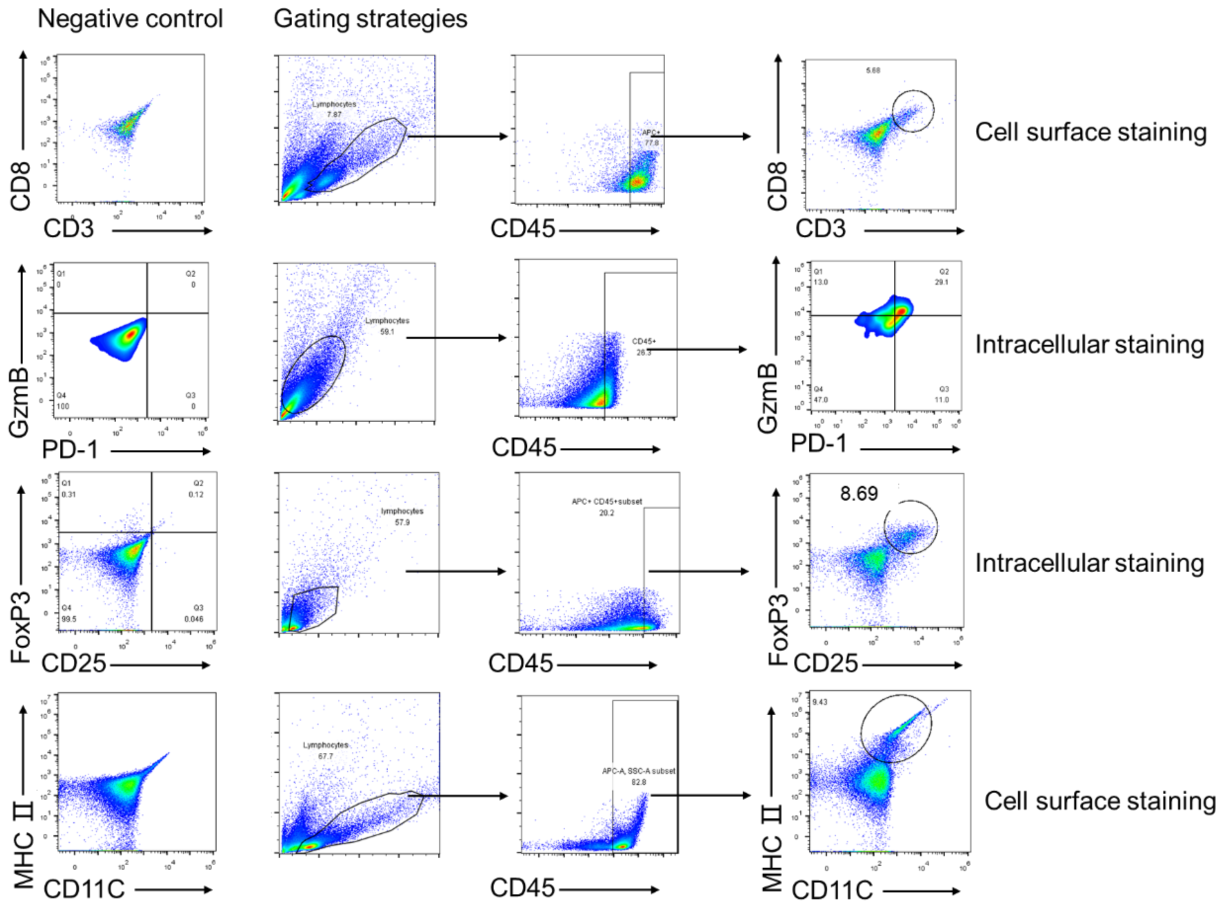

Figure S2. The negative control and gating strategies of FACS analysis.

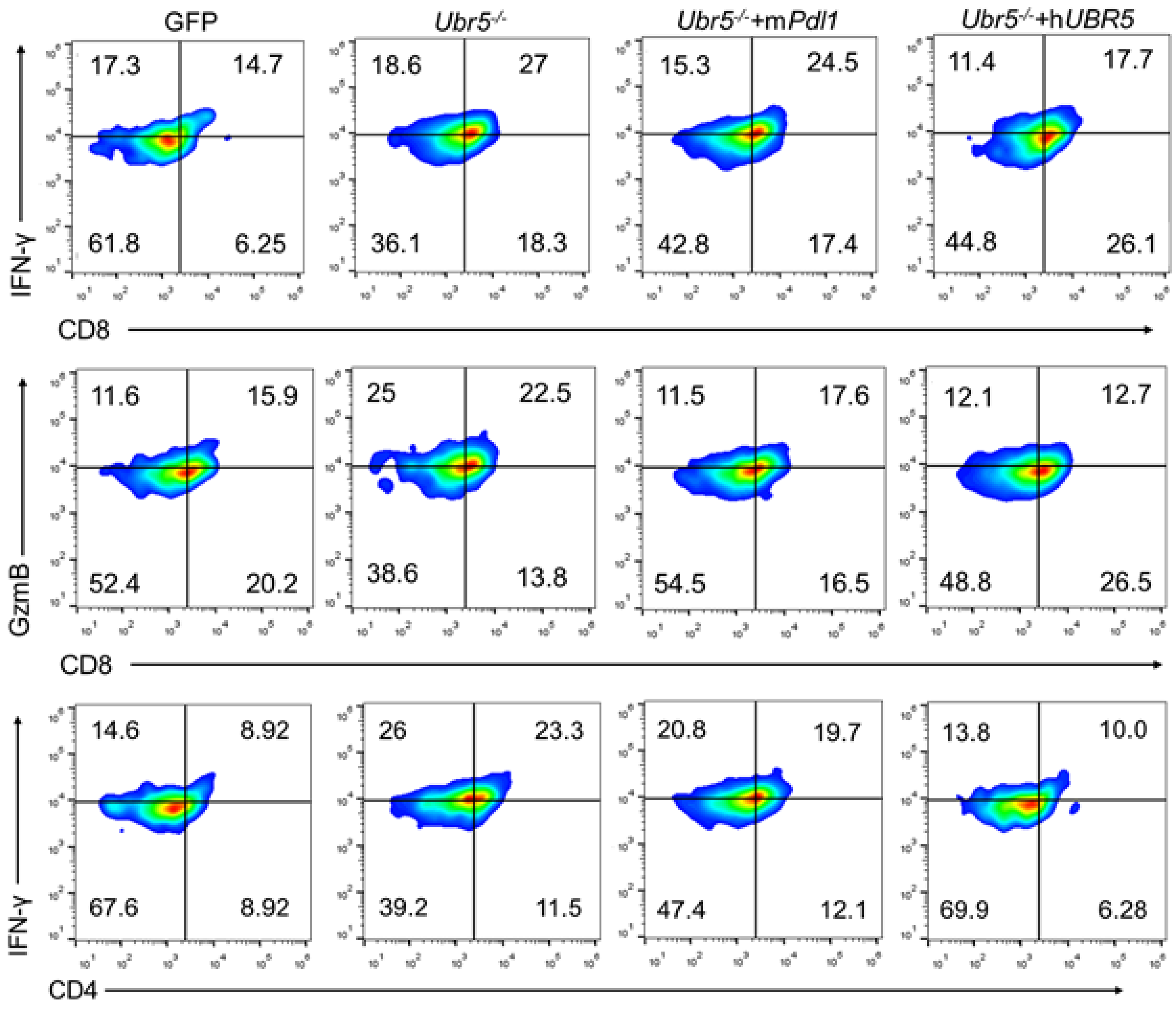

Figure S3. Lower levels of IFN-γ and GzmB were produced by infiltration T cells in tumors of mice bearing h*UBR5* and m*Pdl1*-reconstituted *Ubr5*$^{-/-}$ 4T1 tumor than in *Ubr5*$^{-/-}$ 4T1 tumor.

The percentage of IFN-γ and GzmB producing by CD8^{+} T cells and IFN-γ producing by CD4^{+} T cells were analyzed by flow cytometry in tumors of mice bearing WT, *Ubr5*$^{-/-}$, h*UBR5* or m*Pdl1*-reconstituted *Ubr5*$^{-/-}$ 4T1 tumors.

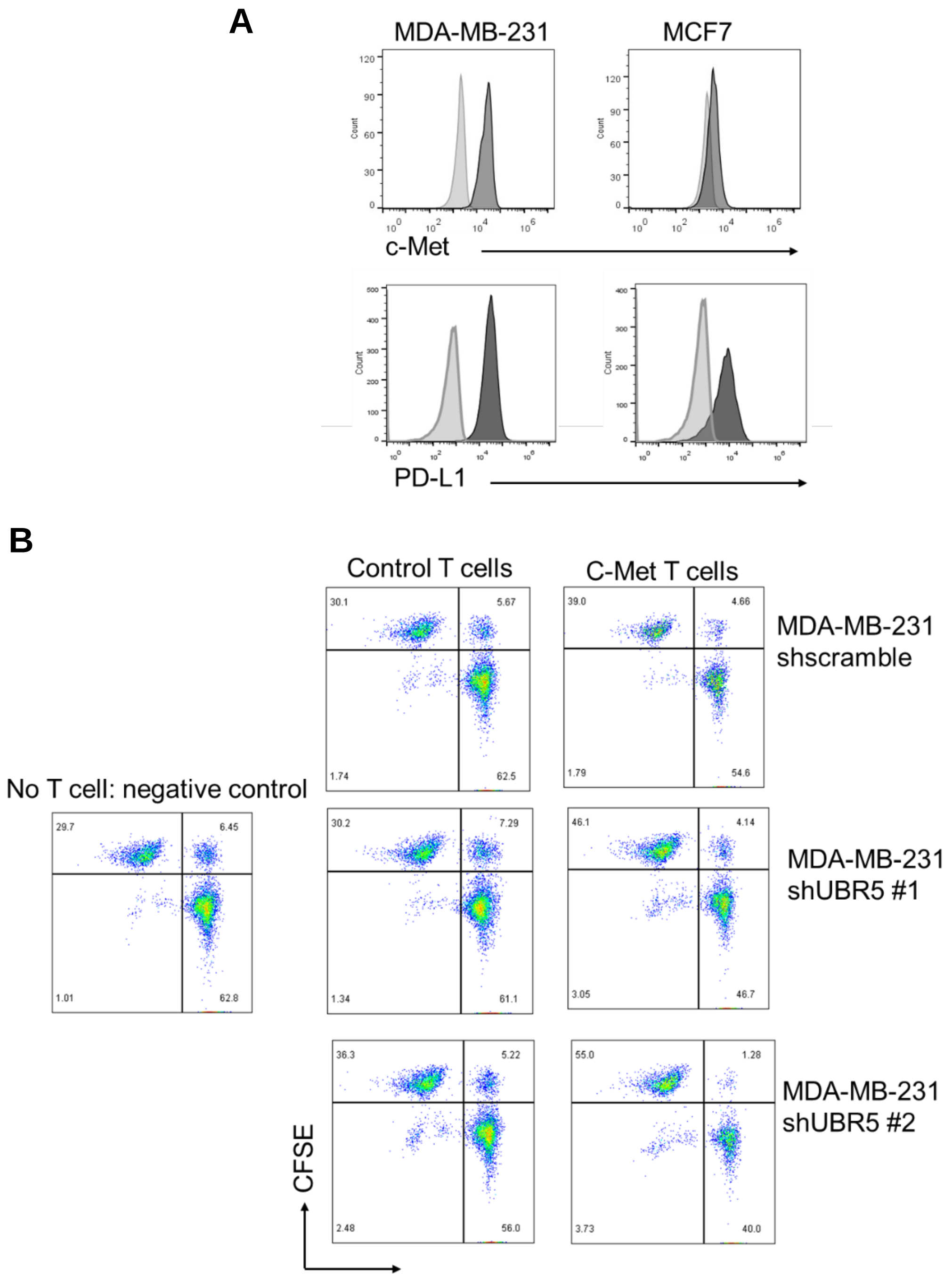

Figure S4. UBR5 deficient decreased PD-L1 expression levels in MDA-MB-231 cells and could promote c-Met specific chimeric antigen receptor T cells-mediated killing efficiency.

(A) The expression of c-Met and PD-L1 in MDA-MB-231 and MCF cells were evaluated by flow cytometry. (B) T cells Cytotoxicity difference toward BT549 cells with different UBR5 expression levels. dt-Tomato Red stably expressed MDA-MB-231 cells (target cells) were mixed with CFSE labeled MCF7 cells (non-target cells) at a ratio of 1:1, and then co-cultured for 18 h with either control or c-Met specific chimeric antigen receptor T cells at a ratio of 1:2 separately. Cells were harvested and analyzed by flow cytometry. The data are presented as the mean ± SEM (error bar) from three replicates. *P< 0.05.

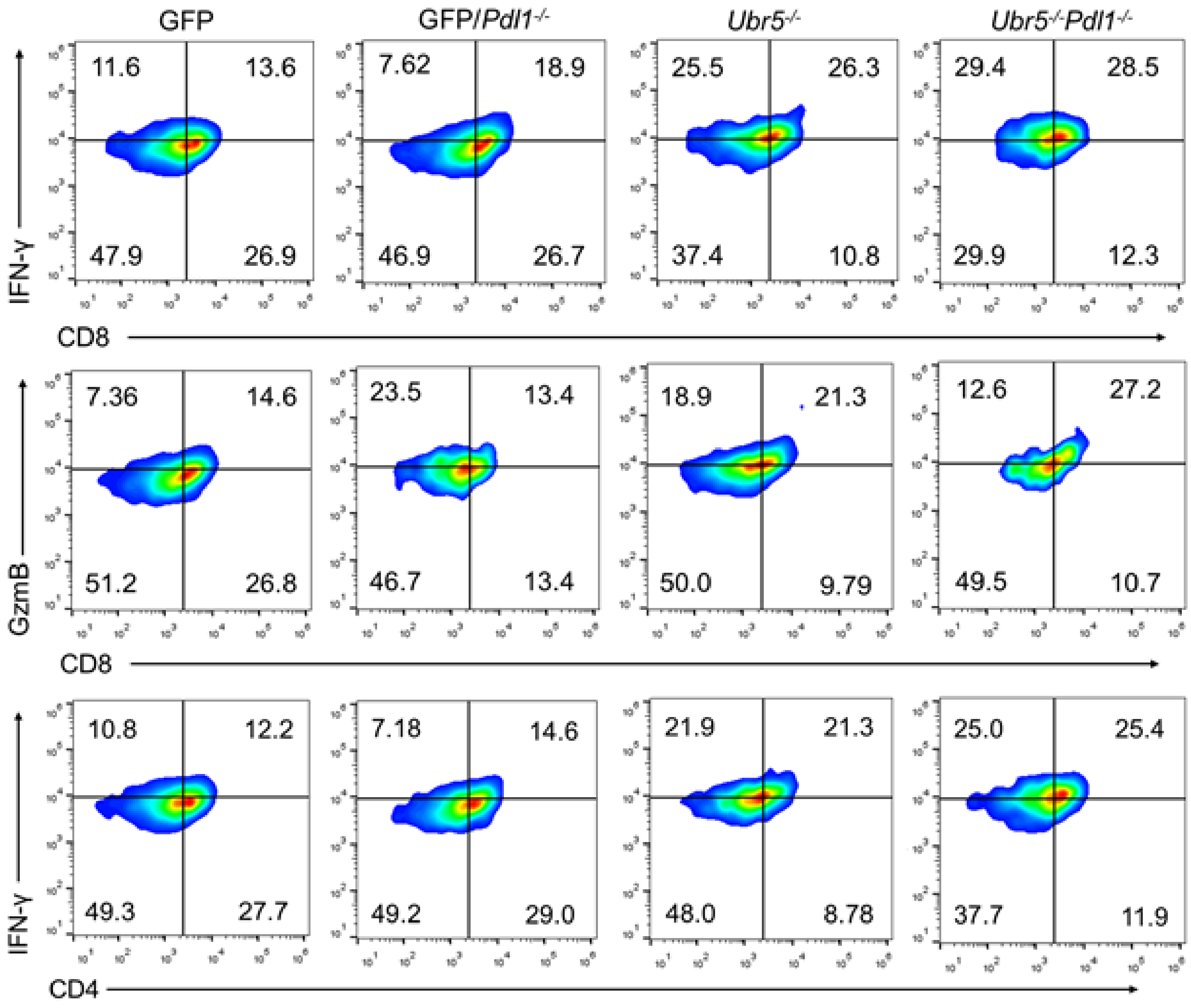

Figure S5. More IFN-γ and GzmB were produced by infiltration T cells in tumors of mice bearing *$Ubr5^{-/-}Pdl1^{-/-}$* 4T1 tumor than in WT, *$Pdl1^{-/-}$*, *$Ubr5^{-/-}$* 4T1 tumor.

The percentage of IFN-γ and GzmB producing by $CD8^{+}$ T cells and IFN-γ producing by $CD4^{+}$ T cells were analyzed by flow cytometry in tumors of mice bearing WT, *$Ubr5^{-/-}$*, *$Pdl1^{-/-}$*, *$Ubr5^{-/-}$ $Pdl1^{-/-}$* 4T1 tumors.

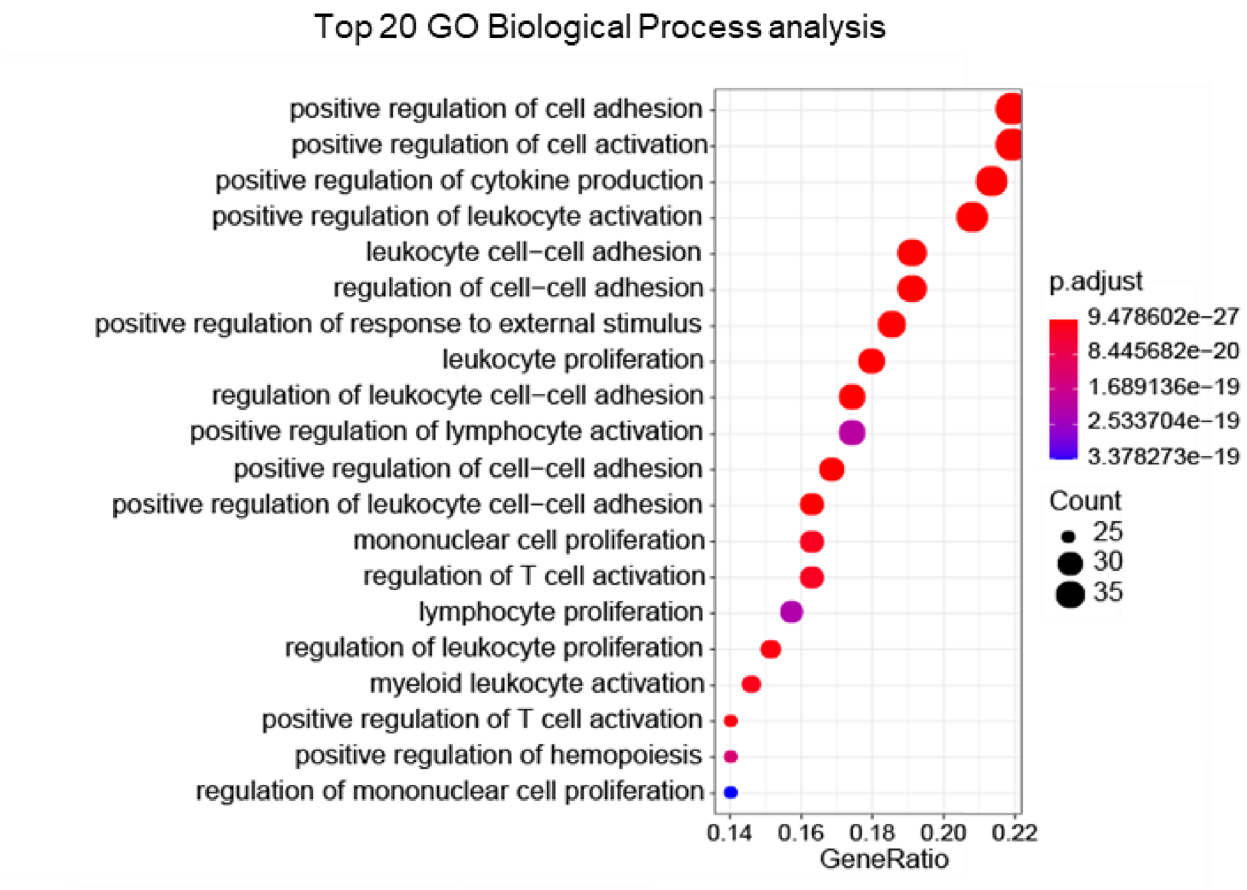

Figure S6. Gene Ontology (GO) analysis of the biological process between GFP and *Ubr5*$^{-/-}$ 4T1 cells treated with IFN-γ.

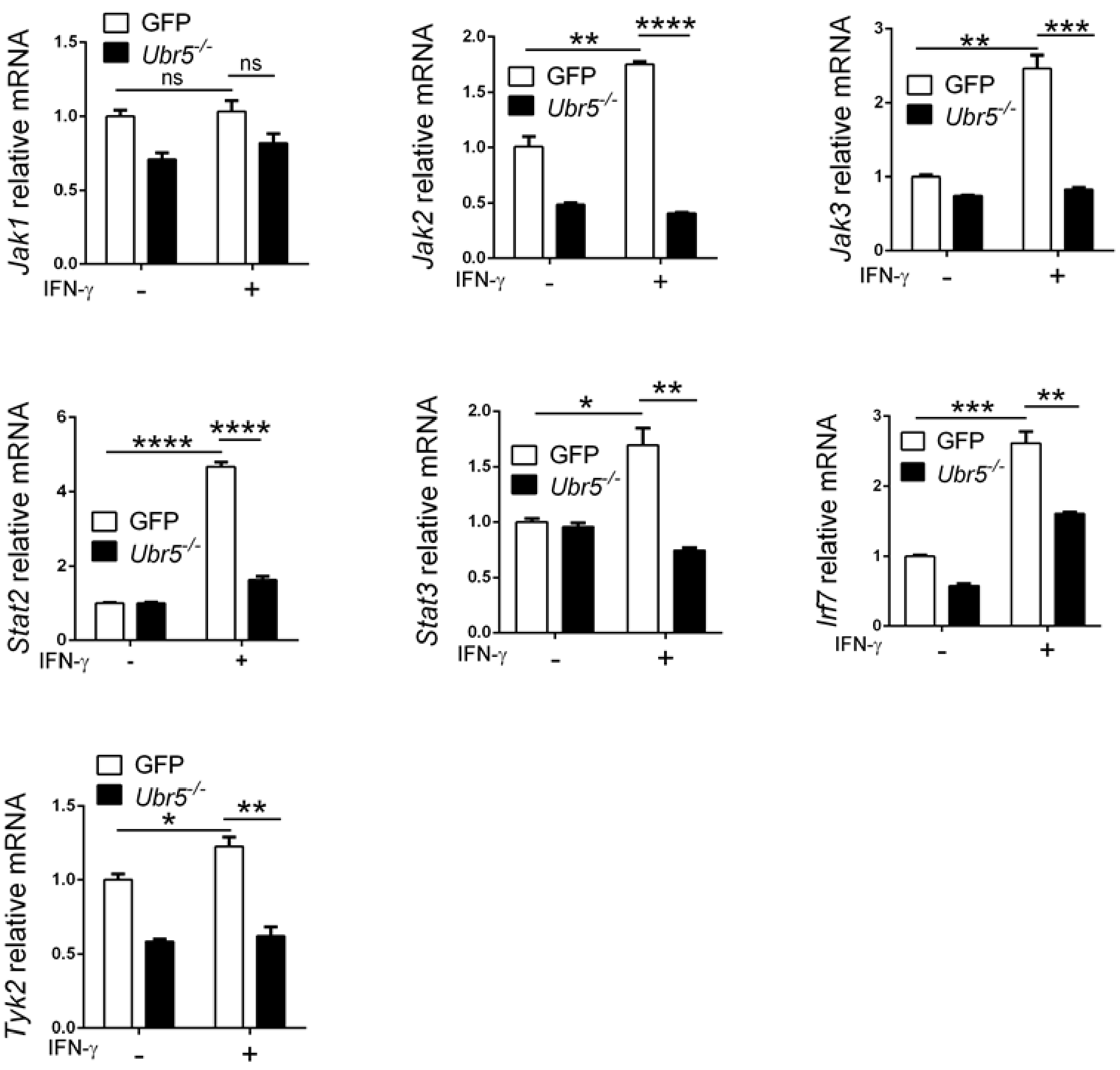

Figure S7. Relative mRNA levels of *Jak1/2/3, Stat2/3, Irf7* and *Tyk2* in GFP and *Ubr5*$^{-/-}$ cells treated with or without IFN-γ.

The mRNA levels of *Jak1/2/3, Stat2/3, Irf7* and *Tyk2* in GFP and *Ubr5*$^{-/-}$ 4T1 cells (treated with or without IFN-γ) were detected. GAPDH was used for normalization. Results are presented as mean ± SEM of three individual experiments. ns, no significance, *P<0.05, **P<0.01, ***P< 0.001, ****P < 0.0001.

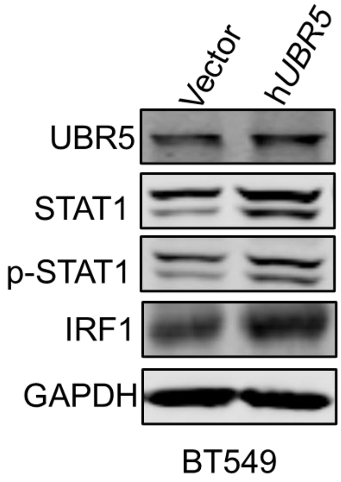

Figure S8. Overexpression UBR5 increased the protein levels of STAT1, pSTAT1 and IRF1 in BT549 cells.

BT549 cells were transfected with either an empty vector or UBR5 plasmids. 24 hours later, the cells were treated with IFN-γ for 24 h. Then, the protein levels of STAT1, pSTAT1 and IRF1 were measured by western blot.

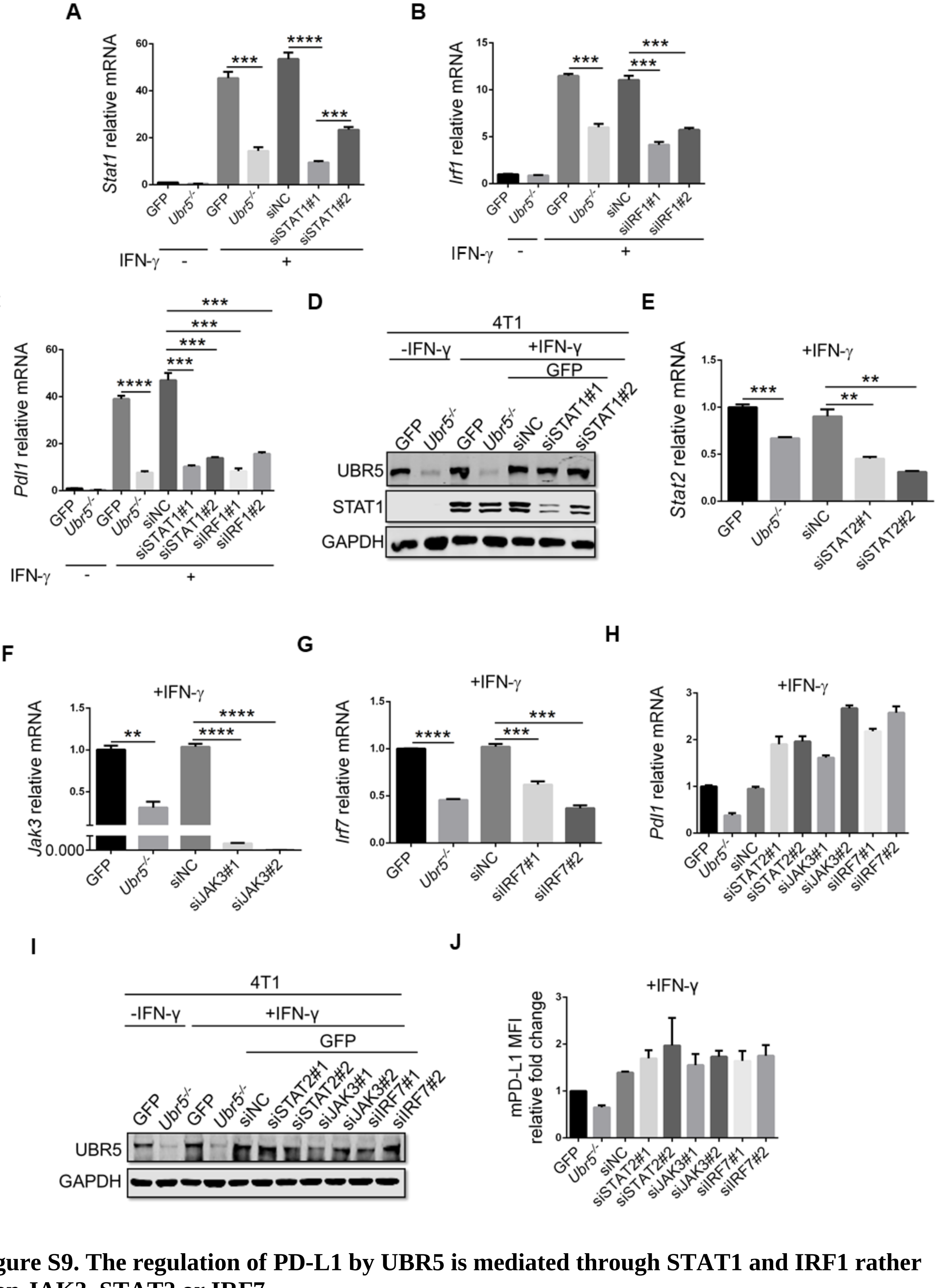

Figure S9. The regulation of PD-L1 by UBR5 is mediated through STAT1 and IRF1 rather than JAK3, STAT2 or IRF7.

(A-D) The GAPDH normalized mRNA levels of *Stat1* (A), *Irf1* (B) and *Pdl1* (C), protein levels of UBR5 and STAT1 (D) were detected separately in siSTAT1 (A) or siIRF1 (B) transiently transfected 4T1/GFP cells. GFP and *Ubr5*$^{-/-}$ 4T1 treated with or without IFN-γ were used for positive and negative controls. siNC transfection served as silencing control. (E-J) The mRNA levels of *Stat2* (E), *Jak3* (F), *Irf7* (G) and *Pdl1* (H), protein of UBR5 (I), and surface PD-L1 level (J) were detected in separately in siSTAT2, siJAK3, siIRF7 transiently transfected 4T1/GFP cells. GFP, *Ubr5*$^{-/-}$ 4T1 cells with IFN-γ stimulation were used for positive and negative controls. GAPDH was used for normalization. Results are presented as mean ± SEM of three individual experiments. ***P< 0.001, ****P < 0.0001.

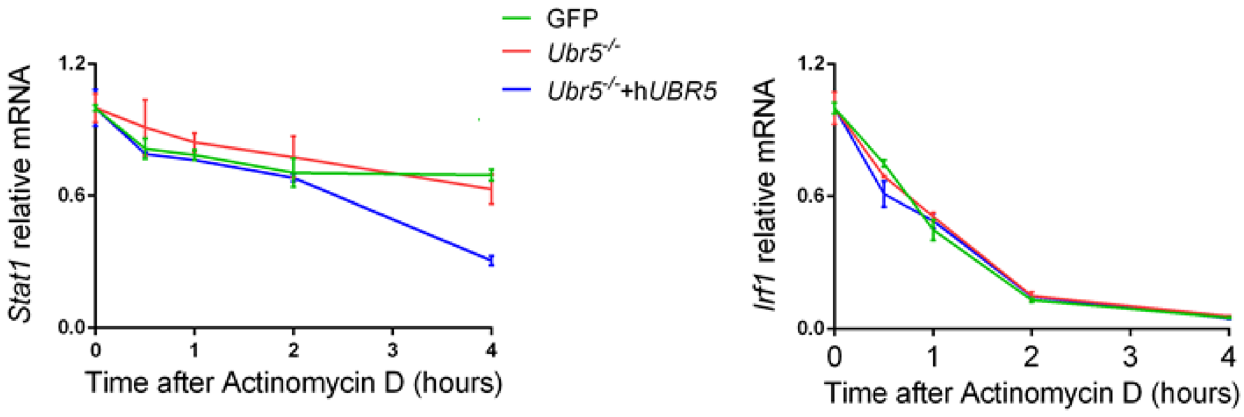

Figure S10.The regulation of *STAT1* and *IRF1* by UBR5 does not depend on mRNA stability.

IFN-γ-treated WT, *Ubr5*$^{-/-}$, and h*UBR5*-reconstituted *Ubr5*$^{-/-}$ 4T1 cells were treated with the transcription inhibitor actinomycin D (1 µg/mL). *Stat1 and Irf1* mRNA levels were quantified using qPCR (±SEM, n = 3). GAPDH was used for normalization. The results are presented as the mean ± SEM from three repliations.

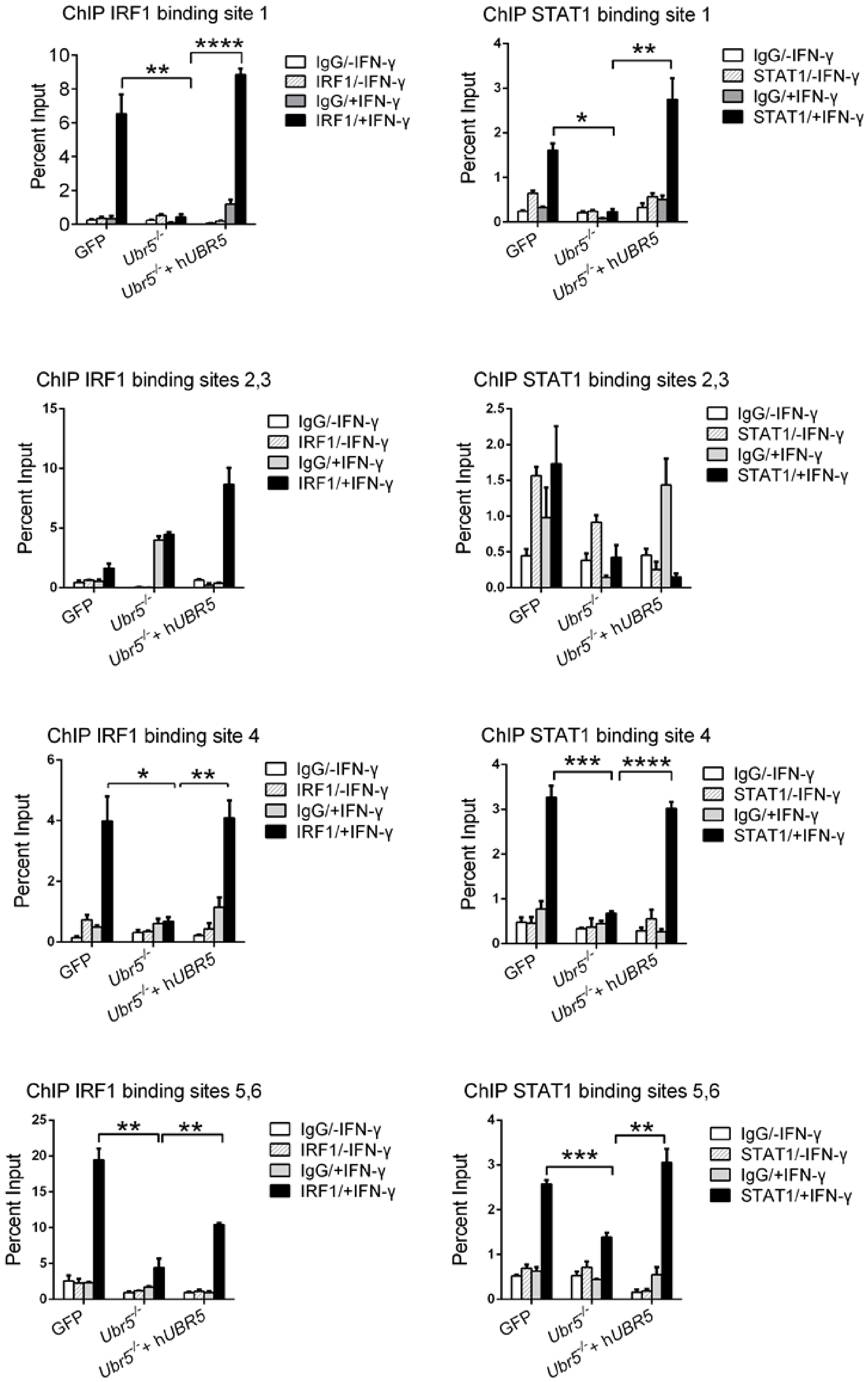
ChIP IRF1 binding site 1
Percent Input
IgG/-IFN-γ
IRF1/-IFN-γ
IgG/+IFN-γ
IRF1/+IFN-γ
GFP
Ubr5-/-
Ubr5-/- + hUBR5
ChIP STAT1 binding site 1
IgG/-IFN-γ
STAT1/-IFN-γ
IgG/+IFN-γ
STAT1/+IFN-γ
ChIP IRF1 binding sites 2,3
ChIP STAT1 binding sites 2,3
ChIP IRF1 binding site 4
ChIP STAT1 binding site 4
ChIP IRF1 binding sites 5,6
ChIP STAT1 binding sites 5,6

Figure S11. The enrichment of STAT1 and IRF1 in the *Pdl1* promoter region is affected by the expression of UBR5.

ChIP assay was performed using anti-IRF1 and STAT1 antibodies in WT, *Ubr5*$^{-/-}$ or h*UBR5*-reconstituted *Ubr5*$^{-/-}$ 4T1 cells after treatment with or without IFN-γ.

Results are presented as mean ± SEM of three replications. *P< 0.05, **P < 0.01, ***P< 0.001, ****P < 0.0001.

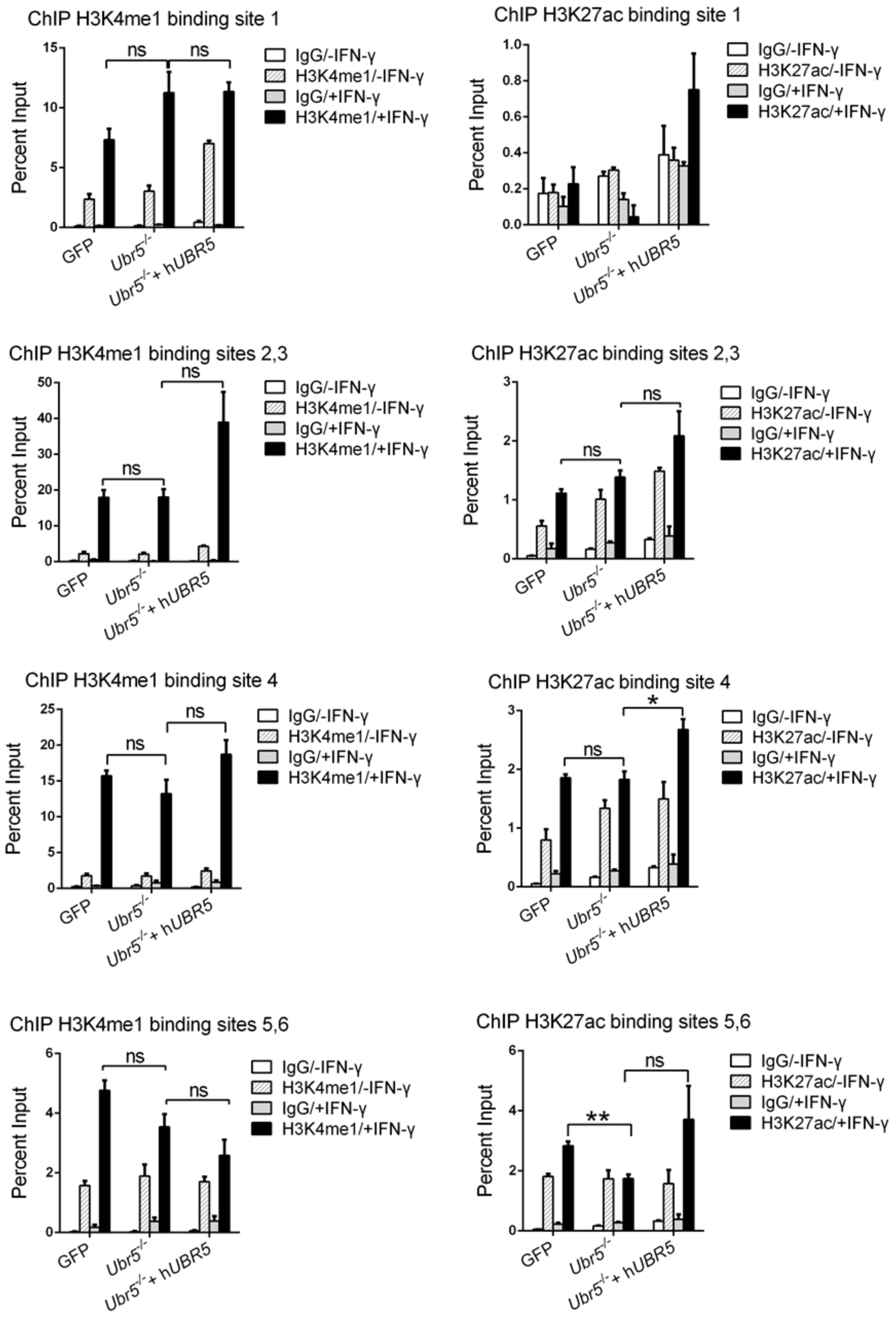
ChIP H3K4me1 binding site 1
ChIP H3K27ac binding site 1
ChIP H3K4me1 binding sites 2,3
ChIP H3K27ac binding sites 2,3
ChIP H3K4me1 binding site 4
ChIP H3K27ac binding site 4
ChIP H3K4me1 binding sites 5,6
ChIP H3K27ac binding sites 5,6
Percent Input
IgG/-IFN-γ
H3K4me1/-IFN-γ
IgG/+IFN-γ
H3K4me1/+IFN-γ
H3K27ac/-IFN-γ
H3K27ac/+IFN-γ
GFP
Ubr5-/-
Ubr5-/- + hUBR5
ns
*
**

Figure S12. The methylation of H3K4 and acetylation of H3K27 are not involved in *PDL1* transcription regulation by UBR5.

ChIP assay was performed using anti-H3K4me1 and H3K27ac antibodies in WT, *Ubr5*$^{-/-}$ or h*UBR5*-reconstituted-*Ubr5*$^{-/-}$ 4T1 cells after treated with or without IFN-γ.
Results are presented as mean ± SEM of three replications. ns, no significance, *P<0.05, **P<0.01.

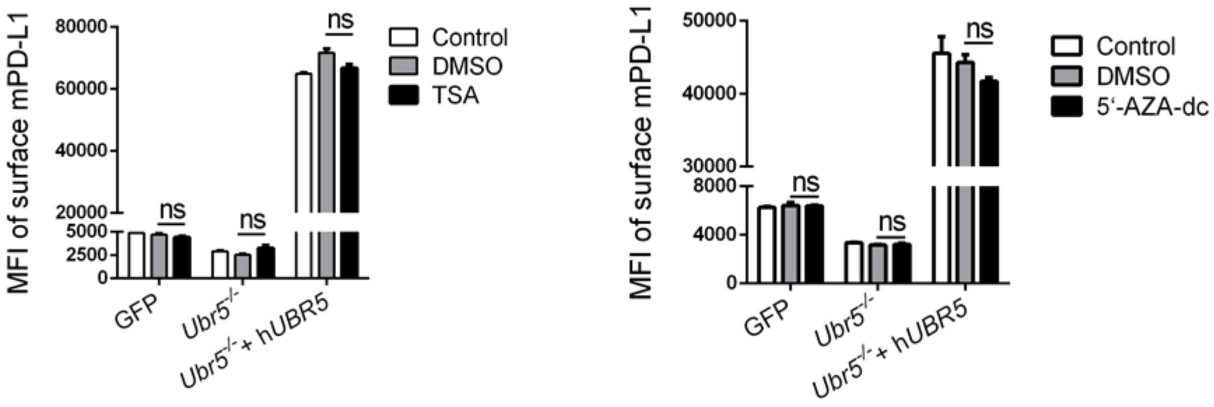

Figure S13. Histone acetylation and methylation are not involved in *PDL1* transcription regulation by UBR5.

IFN-γ-pretreated WT, *Ubr5*$^{-/-}$ and hUBR5-reconstituted *Ubr5*$^{-/-}$ 4T1 cells were treated with 150 nmol/L trichostatin A (TSA), a histone deacetylase (HDAC) inhibitor, for 24 hours (left panel) or 2 mmol/L 5-aza-2'-deoxycytidine (5'-AZA-dC), a DNA methylation inhibitor, for 48 hours (right panel). Surface PD-L1 levels were measured by FACS. The results are presented as the mean ± SEM from three individual experiments.

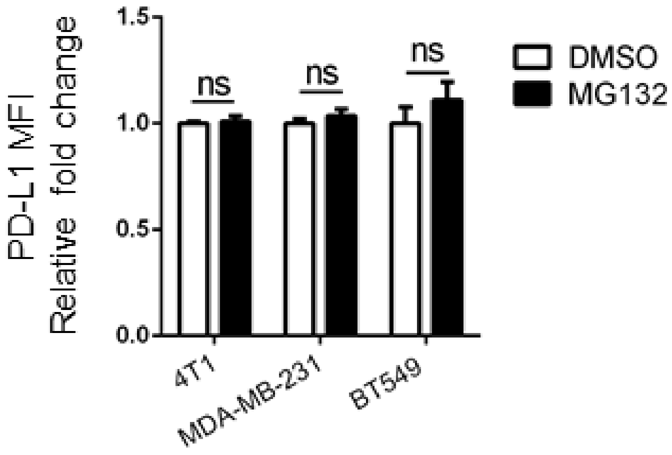

Figure S14. The expression of PD-L1 surface protein is not influenced by UBR5 in the degradation stage.

IFN-γ pre-treated 4T1, MDA-MB-231 and BT549 cells were treated with DMSO or 10 μmol/L MG132 for 6 h. Surface PD-L1 level was measured by FACS.

Results are presented as mean ± SEM of three individual experiments. ns, no significance.

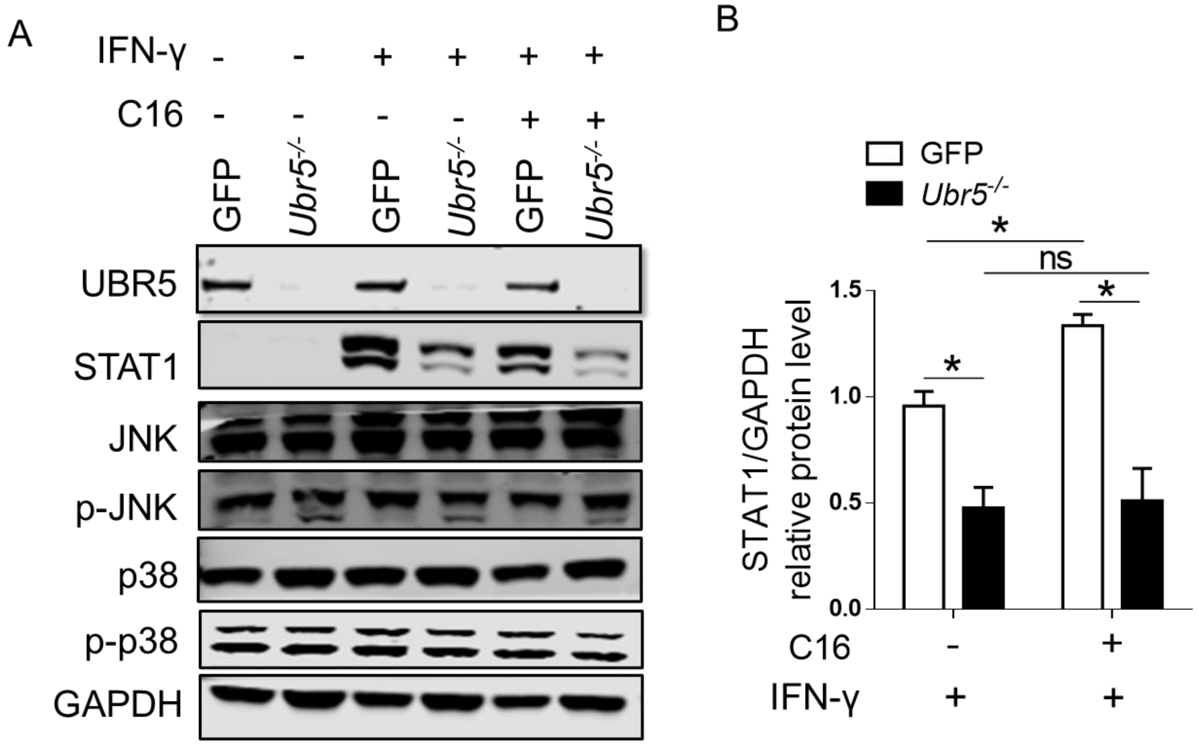

Figure S15. The regulatory activity of UBR5 on STAT1 is no difference when the kinase activity of PKR is inhibited.

(A)The protein levels of STAT1, p38 and JNK (and their phosphorylation form) were detected in GFP and *Ubr5*$^{-/-}$ 4T1 cells that were pre-treated with 0.5 μM PKR inhibitor C16 for 4 h, followed with IFN-γ stimulation 24 h. (B) The quantitative results of STAT1 protein levels are relative to GAPDH with or without C16 treatment in GFP and *Ubr5*$^{-/-}$ 4T1 cells in the presence of IFN-γ.

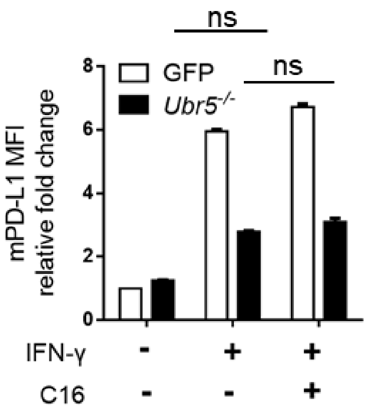

Figure S16. PKR participates in UBR5-mediated *PDL1* transcription activation in a kinase-independent manner.

Surface PD-L1 levels were detected in GFP and *Ubr5*$^{-/-}$ 4T1 cells pretreated with 0.5 µM C16 (PKR inhibitor) for 4 h and then stimulated or not stimulated with IFN-γ for 24 h.
The results are presented as the mean ± SEM from three individual experiments. ns, no significant.

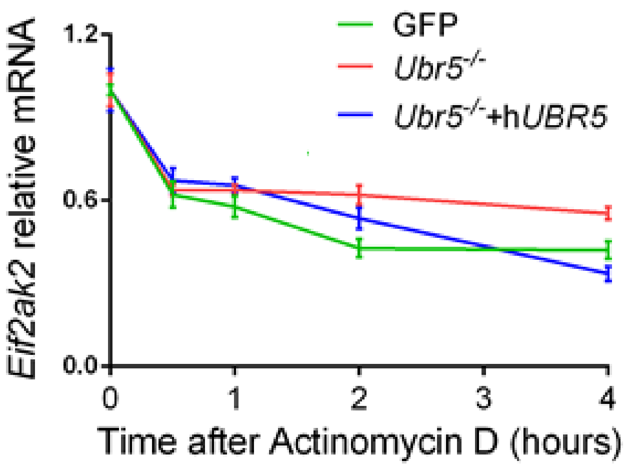

Figure S17. The stability of *Eif2ak2* mRNA is not influenced by UBR5.

IFN-γ-pretreated WT, *Ubr5*$^{-/-}$, and h*UBR5*-reconstituted *Ubr5*$^{-/-}$ 4T1 cells were treated with the transcription inhibitor actinomycin D (1 μg/mL). *Eif2ak2* mRNA levels were quantified using qPCR. GAPDH was used for normalization. The results are presented as the mean ± SEM from three replications.

Table S1. **Primers used in plasmids construction**

Gene name	Primer Sequences
mPD-L1-promoter (-2000-+11bp)	Forward (KpnI) : GCCGGTACCCCAAACCACCTCACCTCTTC<br>Reverse (NheI): GCCGCTAGCTTGGGGACCACGATTTCCTG
mIRF1 promoter (-2000-+100 bp)	Forward (KpnI): GGGTACCCCCAGCATTAGGAAATAGAAGC<br>Reverse (NheI): CTAGCTAGCTCAGCTGAAGCCCAGGCAGA
mSTAT1 promoter (-970-+16bp)	Forward (KpnI): GGGGTACCAAAGAAAACTATTCTTTAAAATGTG<br>Reverse (NheI): CTAGCTAGCCATCCTCTGCAGAAAGAACATGCG
mEif2ak2	Forward (AvrII): CCTAGGATGGCCAGTGATACCCCAGG<br>Reverse (SalI): GTCGACCTAACATGTGTTTCTTTTCTT
mPD-L1	Forward (XbaI): GCTCTAGAATGAGGATATTTGCTGGCAT<br>Reverse (EcoRI): CCGGAATTCTTACGTCTCCTCGAATTGTG
hPDL1 promoter (-2000-0 bp)	Forward (KpnI): GGGGTACCTTTCTCTTTTTCTAAACACAGCCTG<br>Reverse (NheI): CTAGCTAGCGGGGCCGCGCGGGACGCGCCAG
hIRF1 promoter (-820-+138bp)	Forward (KpnI): CGGGTACCCGACCTTGAAAACTACTCAGC<br>Reverse (NheI): CTAGCTAGCAAGAGGGAAGAAGGCAGAG
hSTAT1 promoter (-972-+884bp)	Forward (KpnI): GAGGTACCTGTCATGGGAGGAAACTGGTGG<br>Reverse (NheI): CTAGCTAGCCCTTATCTATACAAACAACATTC
UBR5-ΔPABC overlap PCR upstream	Forward (BstBI): GTTCGAAATGATGACGTCCATCCATTTCGT<br>Reverse: ATCAGCTCCATTTTCTCTAAAGGGCCTAGT
UBR5-ΔPABC overlap PCR downstream	Forward: ACTAGGCCCTTTAGAGAAAATGGAGCTGAT<br>Reverse (NotI): AAGCGGCCGCTTACTACACAAAACCAAAATTCTTG
hPD-L1 promoter ΔIRF1 overlap PCR upstream	Forward (SacI): CGAGCTCTAGAAGTTCAGCGCGGGATA<br>Reverse: GTGTATAGAAATGAAGTCCAGTTTTCTTGT

hPD-L1 promoter ΔIRF1- overlap PCR downstream	Forward: ACAAGAAAACTGGACTTCATTTCTATACAC<br>Reverse (BglII): GAAGATCTCAGCGAGCTAGCCAGAGATACT
hPD-L1 promoter ΔSTAT1/3 overlap PCR upstream	Forward (SacI): CGAGCTCTAGAAGTTCAGCGCGGGATA<br>Reverse: TTATCAGAAAGGCGTCTTCAAGGTGACTGA
hPD-L1 promoter ΔSTAT1/3 overlap PCR downstream	Forward: TCAGTCACCTTGAAGACGCCTTTCTGATAA<br>Reverse (BglII): GAAGATCTCAGCGAGCTAGCCAGAGATACT

Table S2. Targeting sequences for gene knockdown or knockout

Gene name	Primer Sequences
shEIF2AK2-1-mus	Forward: TCGAGTGCTGTTGACAGTGAGCGACGCCAGGTTTAACAGCGATTTAGTGAAGCCACAGATGTAAAATCGCTGTTAAACCTGGCG GTGCCTACTGCCTCGGAA<br>Reverse: CGCGTTCCGAGGCAGTAGGCACCGCCAGGTTTAACAGCGATTTTACATCTGTGGCTTCACTAAAATCGCTGTTAAACCTGGCGTCGCTCACTGTCAACAGCAC
shEIF2AK2-2-mus	Forward: TCGAGTGCTGTTGACAGTGAGCGAGGAGTAGCCATTACGTATAAATAGTGAAGCCACAGATGTATTTATACGTAATGGCTACTCCGTGCCTACTGCCTCGGAA<br>Reverse: CGCGTTCCGAGGCAGTAGGCACGGAGTAGCCATTACGTATAAATACATCTGTGGCTTCACTATTTATACGTAATGGCTACTCCTCGCTCACTGTCAACAGCAC
shScramble-homo	Forward: TCGAGTGCTGTTGACAGTGAGCGACCGCAGGTATGCACGCGTTAGTGAAGCCACAGATGTAACGCGTGCATACCTGCGGGTGCCTACTGCCTCGGAA<br>Reverse: CGCGTTCCGAGGCAGTAGGCACCCGCAGGTATGCACGCGTTACATCTGTGGCTTCACTAACGCGTGCATACCTGCGGTCGCTCACTGTCAACAGCAC
shUBR5-1-homo	Forward: TCGAGTGCTGTTGACAGTGAGCGATTGGAACAGGCTACTATTAAATAGTGAAGCCACAGATGTATTTAATAGTAGCCTGTTCCAAGTGCCTACTGCCTCGGAA

	Reverse: CGCGTTCCGAGGCAGTAGGCACTTGGAACAGGCTACTATTAAATACATCTGTGGCTTCACTATTTAATAGTAGCCTGTTCCAATCGCTCACTGTCAACAGCAC
shUBR5-2-homo	Forward: TCGAGTGCTGTTGACAGTGAGCGACAACTTAGATCTCCTGAAATAGTGAAGCCACAGATGTATTTCAGGAGATCTAAGTTGGTGCCTACTGCCTCGGAA<br>Reverse: CGCGTTCCGAGGCAGTAGGCACCAACTTAGATCTCCTGAAATACATCTGTGGCTTCACTATTTCAGGAGATCTAAGTTGTCGCTCACTGTCAACAGCAC
CD274-sgRNA#1	AGGTTTCCTGAACATGCACC
CD274-sgRNA#2	CATACCGTCGTTGCAGTGCT
CD274-sgRNA#3	GACCTAGGTGCCTTTAAGAG

Table S3. Primers used in quantitative RT-PCR (RT-qPCR) assays

Gene name	Primer Sequences
GAPDH (Mus)	Forward: AGGTCGGTGTGAACGGATTTG<br>Reverse: TGTAGACCATGTAGTTGAGGTCA
PD-L1 (Mus)	Forward: AGTATGGCAGCAACGTCACG<br>Reverse: TCCTTTTCCCAGTACACCACTA
UBR5 (Mus)	Forward: GTCCATCCATTTCGTGGTCCA<br>Reverse: GGGTGGCTGTTCAAATTGTACTT
IRF1 (Mus)	Forward: GTTGTGCCATGAACTCCCTG<br>Reverse: GTGTCCGGGCTAACATCTCC
STAT1 (Mus)	Forward: GCTGCCTATGATGTCTCGTTT<br>Reverse: TGCTTTTCCGTATGTTGTGCT
Eif2ak2 (Mus)	Forward: ATGCACGGAGTAGCCATTACG<br>Reverse: TGACAATCCACCTTGTTTTCGT
CD40 (Mus)	Forward: TGTCATCTGTGAAAAGGTGGTC<br>Reverse: ACTGGAGCAGCGGTGTTATG

Siglec15 (Mus)	Forward: ACACCGCTGGCTACTTGG<br>Reverse: GTGTGCTGTGACAAAGGCAG
Isg15 (Mus)	Forward: GGTGTCCGTGACTAACTCCAT<br>Reverse: TGGAAAGGGTAAGACCGTCCT
GAPDH (Homo)	Forward: AATGGACAACTGGTCGTGGAC<br>Reverse: CCCTCCAGGGGATCTGTTTG
UBR5 (Homo)	Forward: CCAGACAGATTGGAATTGGGTAA<br>Reverse: CATGGAGAGTCGCTTGTCCT
PD-L1 (Homo)	Forward: TGGCATTTGCTGAACGCATTT<br>Reverse: TGCAGCCAGGTCTAATTGTTTT
IRF1 (Homo)	Forward: CTGTGCGAGTGTACCGGATG<br>Reverse: ATCCCCACATGACTTCCTCTT
STAT1 (homo)	Forward: CGGCTGAATTTCGGCACCT<br>Reverse: CAGTAACGATGAGAGGACCCT
Eif2ak2 (homo)	Forward: TGGAAAGCGAACAAGGAGTAAG<br>Reverse: CCAAAGCGTAGAGGTCCACTT

Table S4. Primers used in chromatin immunoprecipitation-qPCR assays

Gene name	Primer Sequences
mGAPDH-ChIP	Forward: CCTCTGCGCCCTTGAGCTAGGA<br>Reverse: CACAAGAAGATGCGGCCGTCTC
mPD-L1 promoter binding site-1	Forward: ATTAACTAGAAATATGTTTTGTCT<br>Reverse: AATAGAATAAAGCAATTAAAGT
mPD-L1 promoter binding site-2,3	Forward: AGTGCACTACTTTGGAATAG<br>Reverse: AATGATGTTGAGAAAGACTTTCTGC
mPD-L1 promoter binding site-4	Forward: AACTTTGAGGAAGTCACCAA<br>Reverse: ATATCAAGCAAATGACTCAG
mPD-L1 promoter binding site-5,6	Forward: AATATCCCAAAGCTGACTCT<br>Reverse: TTGGGGACCACGATTTCCTG
